# KIR-HLA interactions extend human CD8^+^ T cell lifespan in vivo

**DOI:** 10.1172/JCI169496

**Published:** 2023-06-15

**Authors:** Yan Zhang, Ada W.C. Yan, Lies Boelen, Linda Hadcocks, Arafa Salam, Daniel Padrosa Gispert, Loiza Spanos, Laura Mora Bitria, Neda Nemat-Gorgani, James A. Traherne, Chrissy Roberts, Danai Koftori, Graham P. Taylor, Daniel Forton, Paul J. Norman, Steven G.E. Marsh, Robert Busch, Derek C. Macallan, Becca Asquith

**Affiliations:** 1 Institute for Infection and Immunity, St George’s, University of London, London, United Kingdom.; 2 Department of Infectious Disease, Imperial College London, London, United Kingdom.; 3 School of Life and Health Sciences, University of Roehampton, London, United Kingdom.; 4 Department of Structural Biology and Department of Microbiology and Immunology, Stanford University School of Medicine, Stanford, California, USA.; 5 Department of Pathology, University of Cambridge, Cambridge, United Kingdom.; 6 Department of Clinical Research, London School of Hygiene and Tropical Medicine, London, United Kingdom.; 7 National Centre for Human Retrovirology, St Mary’s Hospital, Imperial College Healthcare NHS Trust, London, United Kingdom.; 8 Department of Gastroenterology and Hepatology, St George’s University Hospitals NHS Foundation Trust, London, United Kingdom.; 9 Department of Biomedical Informatics and Department of Immunology and Microbiology, University of Colorado School of Medicine, Aurora, Colorado, USA.; 10 Anthony Nolan Research Institute, Royal Free Hospital, London, United Kingdom.; 11 UCL Cancer Institute, UCL, London, United Kingdom.

**Keywords:** Immunology, Adaptive immunity, Genetic variation, T cells

## Abstract

**BACKGROUND:**

There is increasing evidence, in transgenic mice and in vitro, that inhibitory killer cell immunoglobulin-like receptors (iKIRs) can modulate T cell responses. Furthermore, we have previously shown that iKIRs are an important determinant of T cell–mediated control of chronic viral infection and that these results are consistent with an increase in the CD8^+^ T cell lifespan due to iKIR-ligand interactions. Here, we tested this prediction and investigated whether iKIRs affect T cell lifespan in humans in vivo.

**METHODS:**

We used stable isotope labeling with deuterated water to quantify memory CD8^+^ T cell survival in healthy individuals and patients with chronic viral infections.

**RESULTS:**

We showed that an individual’s iKIR-ligand genotype was a significant determinant of CD8^+^ T cell lifespan: in individuals with 2 iKIR-ligand gene pairs, memory CD8^+^ T cells survived, on average, for 125 days; in individuals with 4 iKIR-ligand gene pairs, the memory CD8^+^ T cell lifespan doubled to 250 days. Additionally, we showed that this survival advantage was independent of iKIR expression by the T cell of interest and, further, that the iKIR-ligand genotype altered the CD8^+^ and CD4^+^ T cell immune aging phenotype.

**CONCLUSIONS:**

Together, these data reveal an unexpectedly large effect of iKIR genotype on T cell survival.

**FUNDING:**

Wellcome Trust; Medical Research Council; EU Horizon 2020; EU FP7; Leukemia and Lymphoma Research; National Institute of Health Research (NIHR) Imperial Biomedical Research Centre; Imperial College Research Fellowship; National Institutes of Health; Jefferiss Trust.

## Graphical abstract

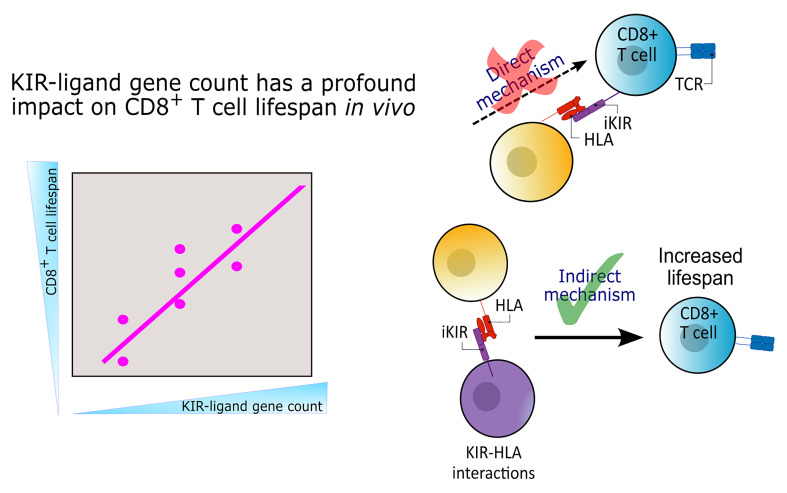

## Introduction

The human killer cell immunoglobulin-like receptors (KIRs) are a family of inhibitory and activating receptors. They are expressed predominantly by NK cells but are also found on T cells in a subset-specific manner (1, 2). Mice have functional analogs (the Ly49 receptors) but no orthologs, and there are significant differences between KIR and Ly49, in both structure and tissue distribution (3). The inhibitory KIRs (iKIRs) are characterized by long cytoplasmic tails, which contain immunoreceptor tyrosine-based inhibitory motifs (ITIMs) that, upon iKIR ligation, become phosphorylated and recruit tyrosine phosphatases (1). The ligands for the iKIR include HLA class I molecules, which iKIRs recognize in broad allotypes, e.g., KIR2DL1 binds HLA C molecules carrying the C2 motif (lysine at position 80) (4–6). The presence of iKIR-ligand pairs is necessary for functional activity; however, iKIRs and their HLA ligands are encoded on different chromosomes and are thus inherited independently. Therefore, within any given individual, not all iKIRs will necessarily have a matching ligand. In an individual carrying a particular iKIR gene as well as an HLA class I allele that can serve as its ligand (i.e., an iKIR-ligand gene pair), that iKIR is referred to as “functional” (e.g., presence of *KIR2DL1* in an individual positive for an *HLA C2* group allele). A “nonfunctional” iKIR is one that is carried in the absence of such a ligand (e.g., the presence of *KIR2DL1* in an individual negative for all *HLA C2* group alleles). Individuals differ in the number of functional iKIR genes they carry, which can vary between 0 and 4. Further complexity is added by allelic polymorphism of KIR genes, which affects protein structure and thus the affinity for HLA class I molecules, as well as protein expression (7).

We have previously found that the number of functional iKIR genes that a person carries significantly impacts CD8^+^ T cell–mediated control of viral infection (8). We investigated 11 well-documented CD8^+^ T cell–mediated HLA class I disease associations in HIV-1, hepatitis C virus (HCV), and human T cell leukemia virus (HTLV-1) infections and found that, in every case, the HLA association was considerably stronger in individuals with a high number of functional iKIR genes than in those with a low number. We showed that this was true for both protective and detrimental HLA class I disease associations. Mathematical modeling showed that this apparently contradictory observation could be explained if iKIR-ligand interactions increased the survival of CD8^+^ T cells (8).

**Figure 1:**
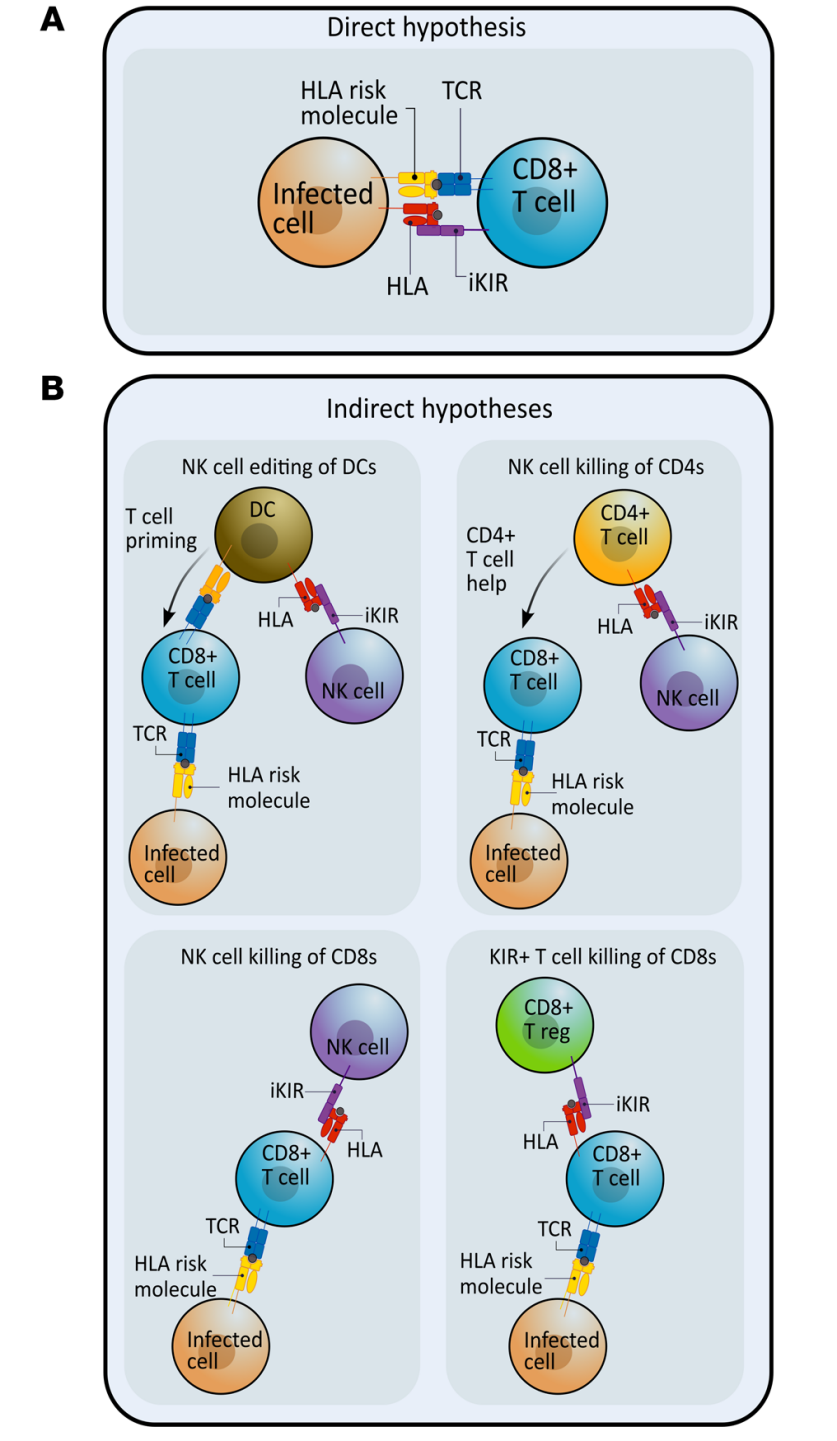
The direct and indirect pathways that could explain iKIR enhancement of CD8^+^ T cell survival. iKIRs (purple) could increase T cell survival and lead to an enhancement of HLA class associations by a number of different pathways. In all diagrams, the HLA class I molecule associated with disease outcome is shown in yellow and is labeled “HLA risk molecule” (interacting with the TCR in blue), and the HLA molecule acting as the iKIR ligand is shown in red. (**A**) Direct hypothesis: iKIR expression on antigen-specific CD8^+^ T cells reduces activation-induced cell death and increases T cell lifespan upon ligation of the cognate KIR ligand. (**B**) Indirect hypotheses: iKIR ligation on other cells can affect CD8^+^ T cell lifespan through a range of mechanisms. (a) NK cells can interact with DCs and shape downstream T cell responses. (b) NK cells can directly kill activated CD4^+^ T cells. (c) Similarly, activated CD8^+^ T cells are also susceptible to NK cell killing. (d) Regulatory KIR^+^ CD8^+^ T cells can kill activated antigen-specific CD8^+^ T cells.

This model prediction is consistent with the growing literature showing that, in iKIR-transgenic mice and in human cells in vitro, iKIR ligation can indeed affect CD8^+^ T cell survival (9–12). The underlying mechanisms can be divided into 2 groups, which we refer to as direct and indirect (Figure 1). Both represent plausible explanations for the enhancement of HLA class I associations by functional iKIR that we have previously observed (8).

Under the direct mechanism (Figure 1A), ligation of iKIRs expressed on the surface of T cells helps to protect that T cell from activation-induced cell death. This phenomenon was demonstrated by Ugolini et al., who showed that, in transgenic mice expressing a human iKIR (KIR2DL3) and its corresponding ligand (HLA-C*03), the iKIR-expressing T cells had a survival advantage that was absent in mice transgenic just for the iKIR or just for the HLA ligand (9). Consistent with this finding, human KIR^+^ T cell clones have been shown to express high levels of the antiapoptotic molecule Bcl2 (13). Furthermore, in vitro coculturing of activated human iKIR–expressing T cell clones with B cell lines expressing the corresponding cognate ligand promoted T cell survival, an effect that was blocked by both iKIR-specific antibodies and HLA class I–specific antibodies (8). However, previous studies suggest that iKIRs are only expressed on a relatively small proportion of predominantly late-stage differentiated T cells (2), raising doubt as to whether such large effects on HLA associations could be achieved through such a small subset of cells.

The indirect mechanisms (Figure 1B) comprise a broader set of pathways, whereby iKIR expression on another cell (e.g., an NK cell or a different T cell) affects the T cell lifespan. Mechanisms within this category include NK cell interactions with DCs, which subsequently affect T cell responses during priming (14), NK killing of activated CD4^+^ and CD8^+^ T cells (10–12), and suppression of T cells by a recently-described population of iKIR-expressing regulatory CD8^+^ T cells (15, 16).

The aim of this study is to address 3 questions: (a) Do iKIRs increase CD8^+^ T cell lifespan in humans in vivo? (b) If iKIRs do increase lifespan, then what is the size of the effect in humans? (c) Are the data most compatible with a direct or indirect mechanism?

Stable isotope labeling is the gold standard for investigating cell dynamics in humans (17–22). Here, we used stable isotope labeling with deuterated water (^2^H_2_O) to quantify the in vivo survival of CD8^+^ T central memory (Tcm) and effector memory T cells expressing CD45RA (Temra) in individuals living with HIV-1, HCV, or HTLV-1 as well as in uninfected controls (17, 23). We found that CD8^+^ T cell survival was significantly affected by the iKIR-HLA genotype (specifically, the number of functional iKIR genes) but not by iKIR expression on the measured T cell. Complementary genetic and immunophenotypic data showed additional evidence that the iKIR-HLA genotype influences immune T cell aging. Our results indicate that iKIRs have a profound impact on T cell survival and are most consistent with an indirect pathway.

## Results

### iKIR expression on CD4^+^ and CD8^+^ T cells is low and not significantly increased by chronic virus infection.

iKIR expression on T cells has previously been shown to be low in healthy individuals (2) but increased in individuals with untreated HIV-1 infection (24). We started by quantifying iKIR expression on T cells from individuals in our cohorts. Our first cohort (cohort 1, who subsequently participated in labeling studies) consisted of individuals with treated HIV-1 infection (all with a low, often undetectable viral load; *n* = 7), individuals with viremic, untreated, chronic HCV infection (*n* = 9), and individuals with chronic HTLV-1 infection (*n* = 3), as well as uninfected individuals (*n* = 4). We quantified the proportion of CD4^+^ and CD8^+^ T cell subsets that expressed KIR2DL1, KIR2DL2/L3, and KIR3DL1 by flow cytometry and identified the factors associated with iKIR expression (see Methods and Supplemental Figure 1; supplemental material available online with this article; https://doi.org/10.1172/JCI169496DS1).

**Figure 2:**
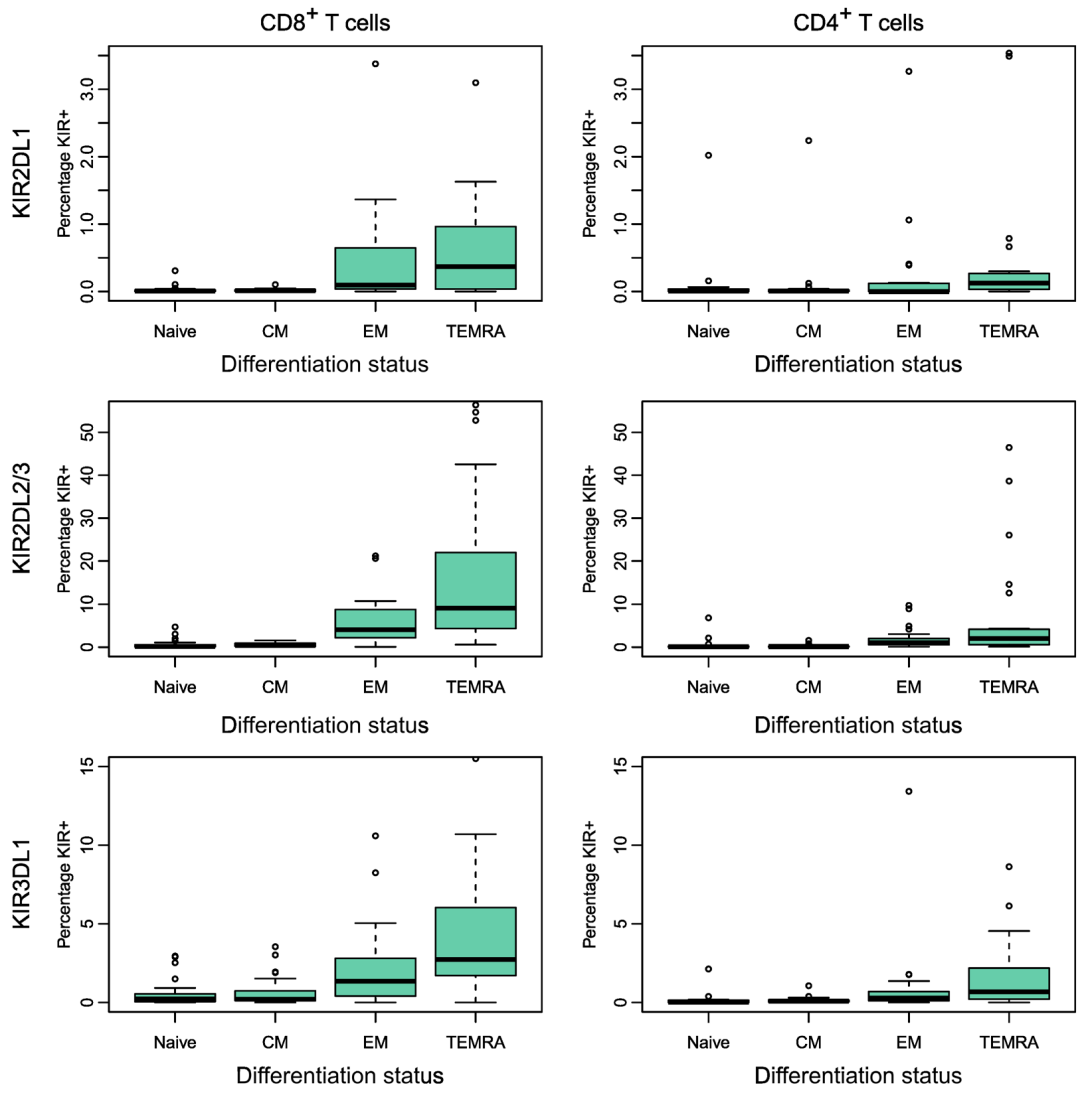
Percentage of T cells expressing different inhibitory KIRs (cohort 1). The percentage of cells in each subpopulation that expressed each of 3 different iKIRs was quantified by flow cytometry for the participants in cohort 1 (*n* = 23). Multivariate regression analysis found that the following were highly significant predictors of increased iKIR expression: a more advanced cell differentiation stage (expressed as an ordinal), *P* = 4 × 10^–14^, CD8 coexpression *P* = 0.0007 and KIR2DL2/L3 *P* = 4 × 10^–12^; male sex was weakly predictive (*P* = 0.049). CMV serostatus and infection status were not significant predictors of iKIR expression. Note that the scale of the *y* axis is different between the rows. Boxes show the median and IQRs. The corresponding data are provided in Supplemental Table 1, and the details of the multivariate regression are given in Supplemental Table 2. *n* = 7 hypotheses tested. *P* value threshold for α = 0.05 is *P* = 0.009 (permutation test).

In general, we observed low iKIR expression across T cell subsets (median 4.7% for CD8^+^ T cells and 0.3% for CD4^+^ T cells), although expression varied markedly among individuals. To identify the determinants of iKIR expression on T cells, we performed multivariate stepwise regression with the following predictors: age, sex, CMV serostatus, cell phenotype (CD4, CD8), T cell differentiation status (as an ordinal), infection status (uninfected, HIV-1, HTLV-1, HCV), and iKIR (KIR2DL1, KIR2DL2/L3, KIR3DL1). We found that iKIR expression increased significantly according to the cell differentiation stage (*P* = 4 × 10^–14^), was higher for KIR2DL2/L3 than for KIR2DL1 (*P* = 4 × 10^–12^), and was higher for CD8^+^ T cells than for CD4^+^ T cells but was not significantly increased by viral infection (either when considered separately or when pooled to increase the power) (Figure 2 and Supplemental Tables 1 and 2). The lack of association between iKIR expression and HIV-1 status and HTLV-1 status was perhaps not unexpected, since the HIV-1–infected participants were aviremic (on antiretroviral treatment), and HTLV-1 infection is known to be largely latent (25). However, we were surprised that iKIR expression by T cells was not elevated in the HCV-infected participants, who were all chronically viremic. To verify this absence of an effect of HCV infection, we recruited a second, larger independent cohort (cohort 2) consisting of 33 uninfected controls and 15 individuals living with HCV and repeated the analysis. The results were very similar to our findings in the first cohort, with the cell differentiation stage (*P* = 4 × 10^–17^), CD8 phenotype (*P* = 0.002), and KIR2DL2/3 (*P* = 7 × 10^–35^) all significant predictors of high iKIR expression. HCV infection and CMV infection remained nonsignificant (*P* = 0.2, *P* = 0.7, respectively) (Supplemental Figure 2 and Supplemental Table 2).

Our results confirm previous findings (2) that only a minority of T cells express iKIR in healthy individuals and extend them, showing that iKIR expression remained low in individuals with chronic viral infection.

### iKIR-ligand genotype strongly determines the T cell lifespan in vivo.

**Figure 3:**
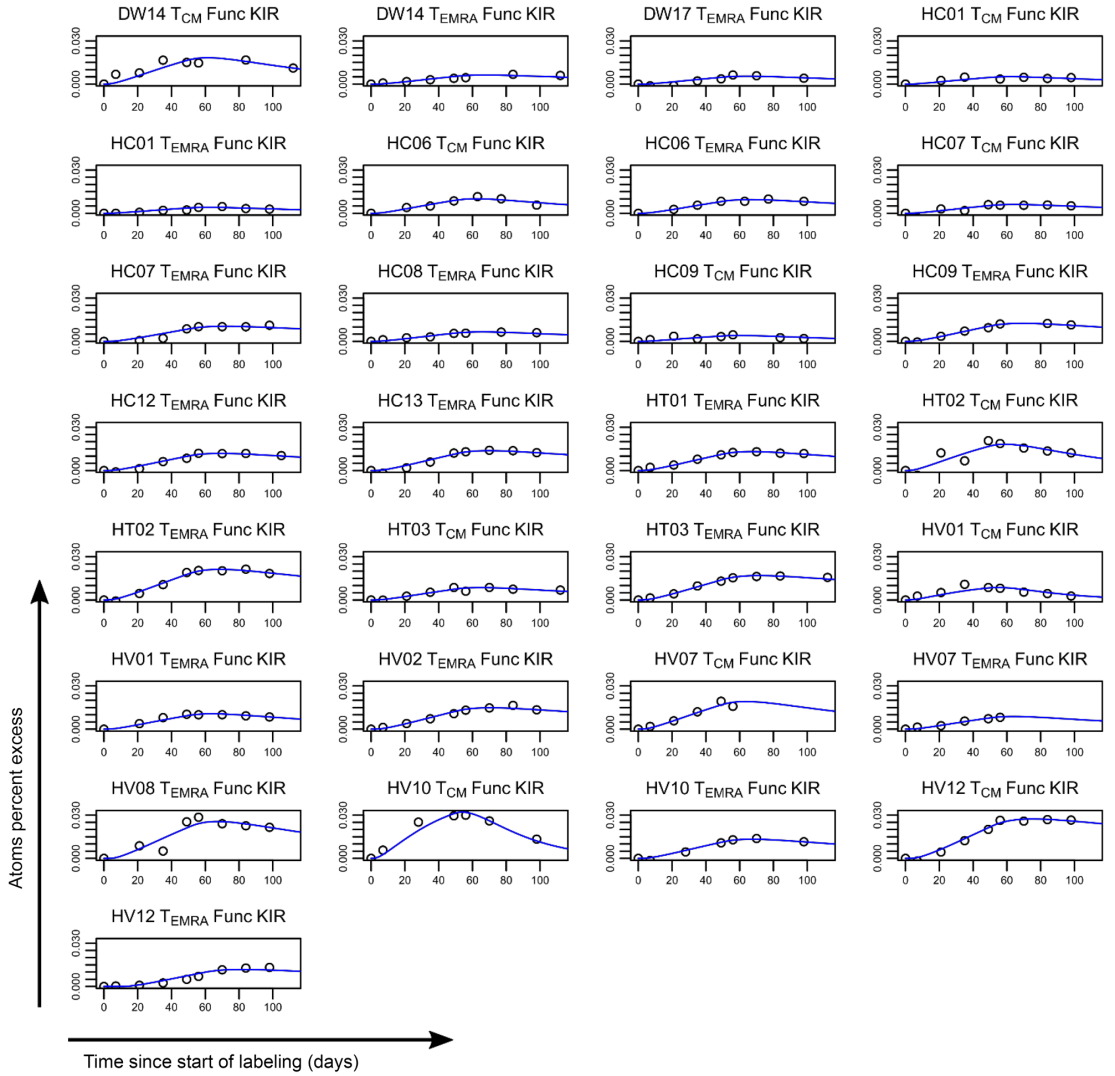
Label enrichment in CD8^+^ T cell subpopulations expressing functional iKIRs. Plots show, for each individual, the label enrichment in the DNA of sorted T cell subpopulations during and following labeling for 49 days. CD8^+^ Tcm and Temra cells were sorted on the basis of their iKIR expression and the individual’s HLA ligand genotype into functional iKIR (Func KIR) (cells expressing an iKIR, in which the individual carried 1 or more allele encoding a ligand); nonfunctional iKIR (Non Func KIR) (cells expressing an iKIR whose ligand is absent from the genome); and KIR^–^ (KIR Neg) (not expressing any of the iKIRs studied). This figure depicts label enrichment in cells expressing functional iKIRs; the remaining data are shown in Figure 4. Circles represent data, and the blue line indicates the best fit of the model to the data. Because of low or absent cell frequencies, it was not possible to collect all cell populations for all individuals.

**Figure 4:**
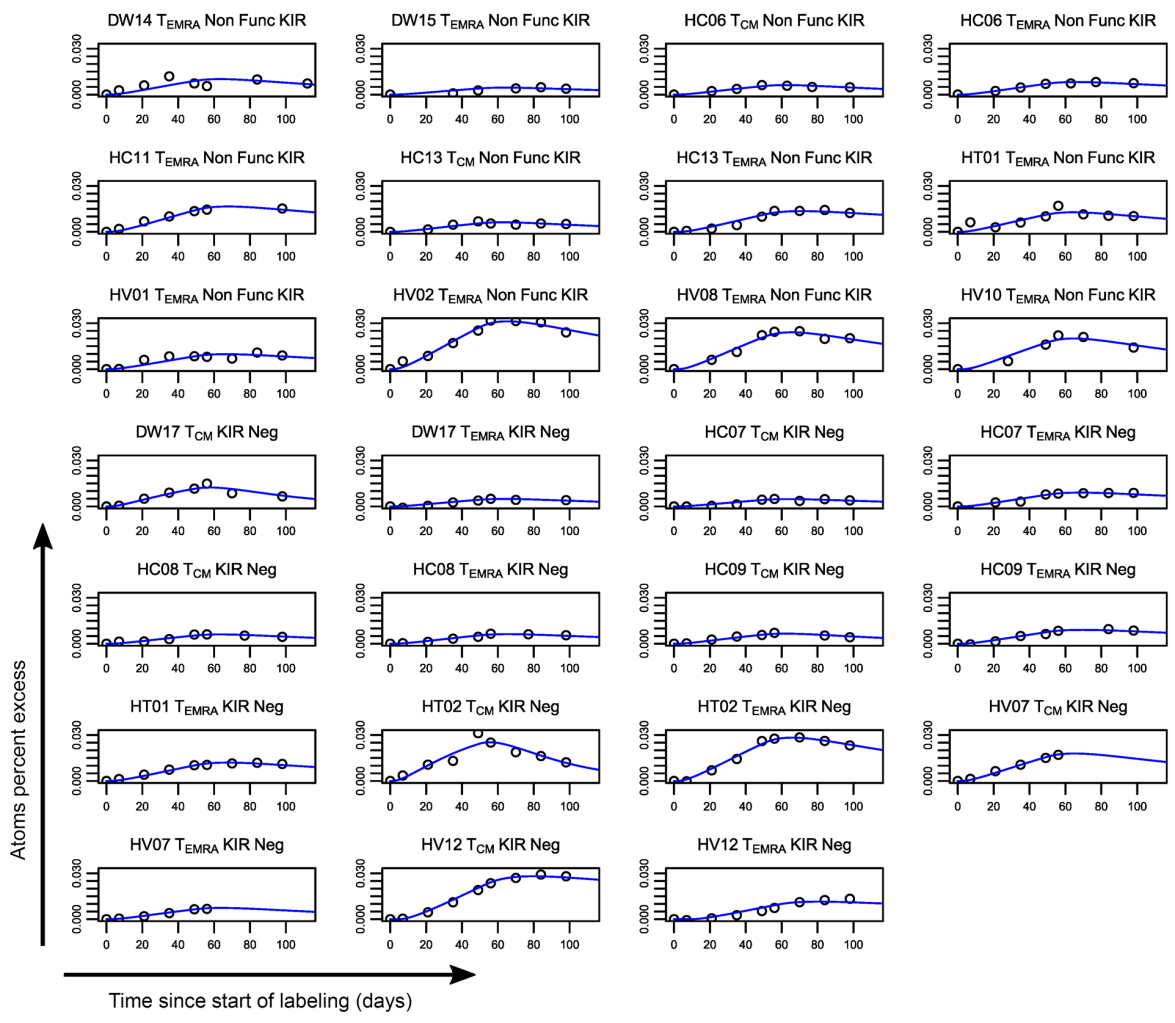
Label enrichment in CD8^+^ T cell subpopulations that only express nonfunctional iKIRs or are iKIR^–^. Plots show, for each individual, the label enrichment in the DNA of sorted T cell subpopulations during and following labeling for 49 days. CD8^+^ Tcm and Temra cells were sorted on the basis of their iKIR expression and the individual’s HLA ligand genotype into functional iKIRs (Func KIR) (expressing an iKIR in an individual carrying 1 or more alleles encoding a ligand), nonfunctional iKIRs (Non Func) (cells expressing an iKIR whose ligand is absent from the genome), and KIR^–^ (KIR Neg) (not expressing any of the iKIRs studied). This figure depicts label enrichment in cells expressing only nonfunctional iKIRs or that were iKIR^–^ (the remaining data are in Figure 3). Circles represent data, and the blue line indicates the best fit of the model to the data. Because of low or absent cell frequencies, it was not possible to collect cells of all populations for all individuals.

To assess the impact of iKIR on CD8^+^ T cell survival in vivo, we used stable isotope labeling. Individuals in cohort 1 (*n* = 23, above) received 7 weeks of deuterated “heavy” water (^2^H_2_O) (see Methods); serial saliva and blood samples were taken during and up to 112 days after the start of labeling. Monocytes were extracted as a rapidly turning over reference population (see Methods and Supplemental Figures 3–5 and Supplemental Table 3). The remaining PBMCs were FACS sorted into CD8^+^ Tcm and CD8^+^ Temra cells on the basis of cell-surface expression of canonical T cell differentiation markers (Tcm: CD45RA^–^CD28^+^; Temra: CD45RA^+^CD28^+^). For all individuals, the KIR and HLA genotype was determined prior to sorting, allowing us to establish bespoke individual gating strategies. This enabled us to further sort both CD8 subsets into cells (Tcm or Temra) expressing functional iKIRs (i.e., iKIRs whose corresponding ligand was encoded by the individual’s HLA alleles) and cells expressing nonfunctional iKIRs (iKIRs in an individual who was negative for all HLA alleles encoding the corresponding ligands). When cells expressing nonfunctional iKIRs were not available (due to low or absent cell frequencies), then cells that were iKIR^–^ were collected instead (Supplemental Figure 1B). Deuterium enrichment in the DNA of monocytes and sorted T cell subpopulations was measured by gas chromatography/mass spectrometry (23). Mathematical models were fitted to the data in order to estimate the lifespan of the different T cell subsets (Figures 3 and 4, Supplemental Table 4, and see Methods).

**Figure 5:**
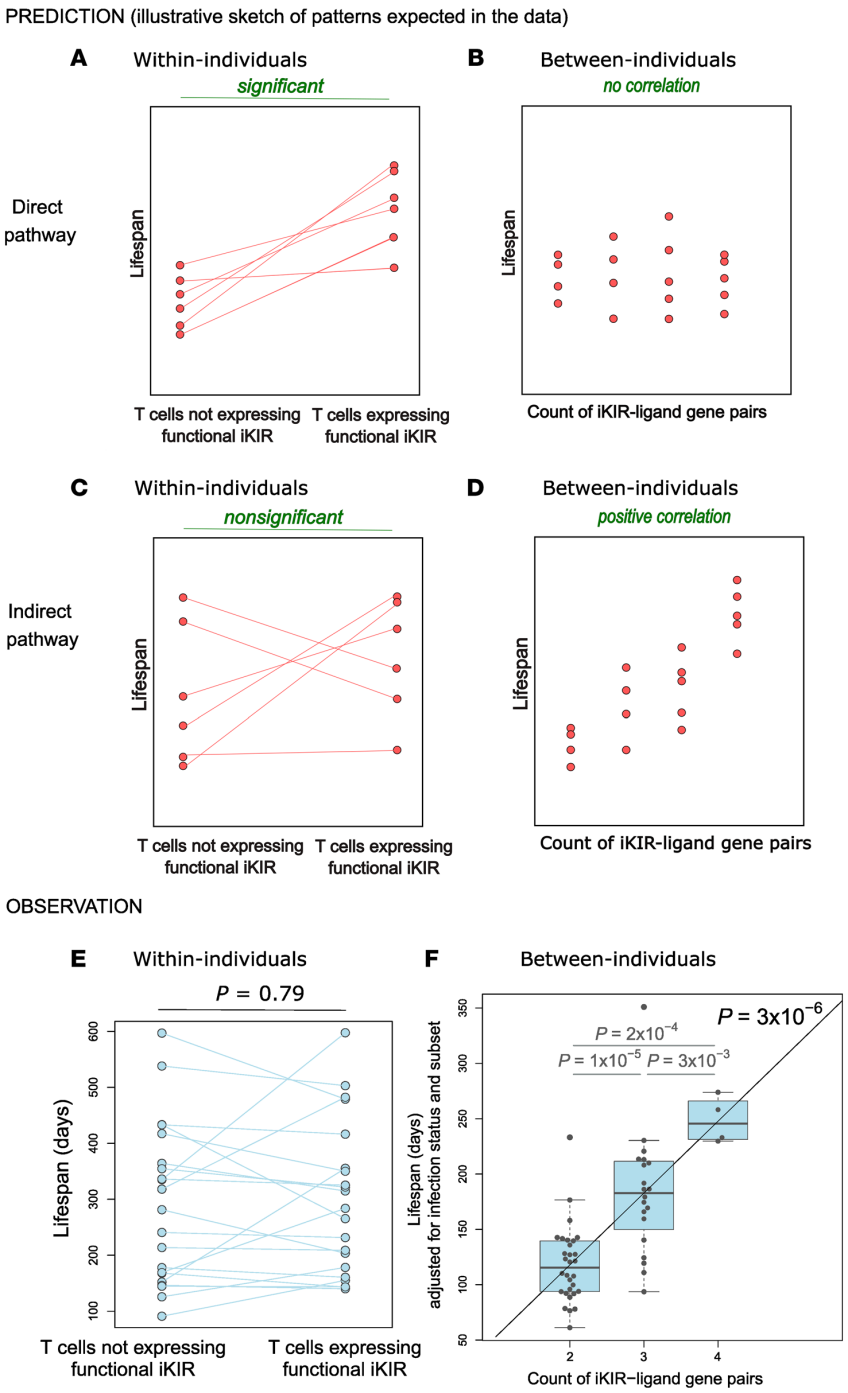
Predicted and observed relationship between iKIRs and T cell lifespan. (**A** and **B**) Sketches of hypothetical data that depict predicted patterns in the data within individuals and between individuals, respectively, if the direct pathway operates. (**C** and **D**) Hypothetical data depicting predicted patterns within and between individuals if the indirect pathway operates. (**E** and **F**) Actual observed results within and between individuals. (**E**) *n* = 21 paired data sets. (**F**) *n* = 53 data points from 18 individuals. Boxes show the median and IQRs with all individual data points superimposed. It can be seen that the observed results are most consistent with the indirect pathway. Note that for the between-individuals comparison, the T cell lifespan has been adjusted (by linear regression coefficients) to allow for infection status of the individual and for cell subpopulation type (Tcm or Temra). This is not necessary for the within-individuals comparison, as this comparison is internally controlled (i.e., both points would be adjusted by the same factor, as both points come from the same individual and the same cell subpopulation (Tem or Temra). We found that the CD8^+^ T cell lifespan was independent of functional iKIR expression (*P* = 0.79, paired Wilcoxon test) and indeed was independent of iKIR expression in general (*P* = 0.50, paired Wilcoxon). In contrast, the CD8^+^ T cell lifespan was significantly determined by iKIR-HLA genotype (*P* = 3 × 10^–6^, multivariate regression).

The aim of the study was 3-fold: (a) To test whether iKIR (expression or genotype) was a determinant of T cell survival; (b) to quantify the impact of iKIR on T cell lifespan; and (c) to determine which of the 2 pathways, direct or indirect, was most likely. The study was designed so that the 2 different pathways (summarized in Figure 1) would give 2 distinct patterns to the data (Figure 5, A–D). If the direct pathway operates, in which iKIRs expressed directly on a CD8^+^ T cell increases that particular T cell’s lifespan when ligated (Figure 1A), then, in a paired, within-individual study, we would predict that the cells expressing functional iKIRs would live longer than cells that were iKIR^–^ or expressing nonfunctional iKIRs (Figure 5A). But in a between-individuals study, we would predict no relationship between the iKIR genotype (specifically the number of iKIR-HLA ligand gene pairs carried by the individual) and T cell lifespan. This is because the data include measurements not just of cells expressing functional iKIRs but also of cells that express no iKIR and cells that express nonfunctional iKIRs, both of which would obscure any signal coming from cells expressing functional iKIR. Furthermore, even within those populations of cells that express functional iKIRs, there is no reason to expect a positive correlation (i.e., given that a cell expresses functional iKIRs, there is no reason to expect the number of functional iKIR genes to affect its lifespan further) (Figure 5B). Conversely, if an indirect mechanism dominates (Figure 1B), then iKIR expression on the measured CD8^+^ T cell would be irrelevant, and thus we would predict that there would be no relationship between functional iKIR expression and cell survival within individuals (Figure 5C), but when we analyze between individuals, we would predict that the number of iKIR-ligand gene pairs carried by the individual would be positively correlated with CD8^+^ T cell lifespan (Figure 5D). Previous work has shown that different CD8 memory/effector T cell subsets have different lifespans (17); thus, in order to rule out confounding effects of memory/effector cell differentiation, we performed the analysis for matched subsets.

### Expression of functional iKIRs is not a significant predictor of CD8^+^ T cell lifespan (within-individual comparison).

First, we investigated whether a CD8^+^ T cell’s lifespan is influenced by its expression of functional iKIRs (i.e., iKIR expression in which the individual carried 1 or more alleles encoding the ligand). We estimated the lifespan of a total of 54 subsets of T cells from 18 individuals (Figures 3 and 4, and Supplemental Table 4) and assessed whether Tcm and Temra expressing functional iKIRs had a longer lifespan than matched Tcm and Temra cells from the same individual expressing only nonfunctional iKIRs or matched Tcm and Temra cells that were iKIR^–^. We found no evidence that T cell lifespan was determined by functional iKIR expression (*P* = 0.79, 2-tailed Wilcoxon; Figure 5E). Repeating the analysis excluding the iKIR^–^ T cells did not change the conclusion (*P* = 0.82, 2-tailed Wilcoxon). Furthermore, a multivariate regression to test whether expression of functional iKIRs predicted the T cell lifespan (with covariates: cell subpopulation [Tcm or Temra], infection status [HIV-1/HCV/HTLV-1/control]) also showed that expression of 1 or more functional iKIRs was not a significant predictor of T cell lifespan (*P* = 0.34, linear multivariate regression, Supplemental Results 1 and Supplemental Table 5A).

### The iKIR-ligand gene pairs count is a highly significant predictor of CD8^+^ T cell lifespan (between-individuals comparison).

Next, we investigated whether, between individuals, an individual’s iKIR and HLA genotype (specifically the count of iKIR–HLA ligand gene pairs in their genome) was a significant predictor of T cell lifespan. In linear multivariate regression (with covariates: cell subpopulation [Tcm or Temra], infection status [HIV-1/HCV/HTLV-1/control]), the iKIR-ligand gene pair count was a significant determinant of T cell survival (Figure 5F) (estimate [Est] = +0.41, *P* = 3.4 × 10^–6^; *n* = 54 T cell subsets from 18 individuals, Supplemental Results 2, Supplemental Table 5B). As well as being highly statistically significant, the size of the effect was striking: in an individual with 2 iKIR-ligand pairs in their genome, after correcting for viral infection and cell phenotype (i.e., for the baseline of Tcm cells in an uninfected individual), their memory CD8^+^ T cells lived, on average, for 125 days; in contrast, in an individual with 4 iKIR-ligand pairs in their genome, their memory CD8^+^ T cells lived for 250 days — a doubling in survival. Inclusion of the covariates age, sex, CMV serostatus, and viral load did not change this conclusion. We had excluded from this analysis any individuals carrying alleles encoding the classical KIR3DL2 ligands (HLA-A*03 and HLA-A*11), as there is evidence that the behavior of KIR3DL2, a framework KIR with an additional ITIM motif in its cytoplasmic tail, might be different from that of the other iKIRs (26–28). When we included these individuals, the overall conclusions were unchanged, but the size of the effect was slightly smaller (Est = +0.38, *P* = 2 × 10^-5^), consistent with the idea that KIR3DL2 is distinct from the other inhibitory KIRs. Previously, we have also used the inhibitory score (the count of functional iKIRs weighted by strength of the KIR-ligand interaction) as a predictor. Here, the count and the score (which are strongly correlated) performed similarly, and there was no significant benefit of one over the other (*P* = 0.09, Davidson-MacKinnon J test, Supplemental Results 3). In contrast, counting only the number of iKIRs, without considering the presence of the ligand, performed significantly worse (*P* = 9 × 10^–5^, Davidson-MacKinnon J test, Supplemental Results 3), indicating that ligation of the iKIR is essential for the increase in T cell lifespan.

Comparing the results (Figure 5, E and F) with the predictions of the 2 different pathways (Figure 5, A–D), it can be seen that the observed impact of iKIR expression and genotype on T cell survival is most consistent with the indirect pathway.

One possible caveat to this conclusion in favor of the indirect pathway was that if cells upregulated and downregulated iKIRs rapidly during the course of the labeling experiment, then the cells sorted ex vivo as expressing functional iKIRs might actually have been iKIR^–^ or expressing nonfunctional iKIRs in vivo and vice versa. This could potentially disguise any association between T cell lifespan and functional iKIR expression between individuals. However, the proportion of Tcm and Temra cells that expressed iKIRs was low (Figure 2). We calculate that, for differences in this small population of T cells (median 6% of Tcm and Temra) to be responsible for the doubling in the bulk T cell lifespan seen between individuals, then iKIR expression would need to increase the survival of this minority cell population by nearly 20-fold (Supplemental Results 4). Furthermore, as argued above, we would not expect the direct pathway to generate a correlation between the functional iKIR count and the CD8^+^ T cell lifespan. In particular, the fraction of iKIR^+^ CD8^+^ T cells would need to be significantly positively correlated with the number of iKIR-ligand gene pairs, which we did not observe (Spearman’s R [Rs] = –0.08, *P* = 0.77, data not shown). Similarly, there was no strong correlation between the fraction of CD8^+^ T cells expressing functional iKIRs and the number of iKIR-ligand gene pairs (Rs = +0.23, *P* = 0.42, data not shown). These observations further support the suggestion that the indirect rather than the direct mechanism is operating.

### iKIR expression and T cell activation.

We also assessed Ki67 expression in cohort 2 as a complementary approach to assess the relationship between iKIR expression and proliferation. We found significantly higher levels of Ki67 expression in iKIR^+^ T cell subsets when compared with the corresponding iKIR^–^ cells, regardless of whether or not the iKIR was functional (Supplemental Figure 6). We considered 2 possible explanations for this observation. The first is that iKIR^+^ cells were dividing more rapidly than iKIR^–^ cells, and the second is that iKIR expression was upregulated transiently in proliferating/activated cells. The first interpretation is inconsistent with the in vivo labeling data. We therefore explored the relationship between iKIR expression and T cell activation in vitro. We showed that iKIR expression was indeed significantly upregulated on CD8^+^ T cells following stimulation with anti-CD3/anti-CD28 (αCD3αCD28), that iKIR expression was higher on activated (CD38^+^) cells than on quiescent cells (CD38^–^), and that iKIR expression was stable at a cellular level for at least 72 hours (Supplemental Figures 7–9. This is consistent with work showing upregulation followed by slow downregulation of iKIRs on human T cell clones following activation (29).

### Relationship between the iKIR-ligand gene pair count and CD57 expression.

Intuitively, one would expect that in individuals with a high number of iKIR-HLA ligand pairs (i.e., a high functional iKIR gene count), in which the survival of CD8^+^ T cells is elevated, the average age of a CD8^+^ T cell would be increased. This intuition was confirmed by mathematical modeling, which predicted a weak relationship between cell survival (and thus the iKIR/HLA genotype) and cell age (defined as the time between a cell entering the memory compartment and sampling; see Methods). To test this prediction, we first looked for a cell phenotype that is correlated with cell age. It has been reported that expression of CD57, a terminally sulfated glycan carbohydrate epitope, is increased as cells age, although it is not a marker of replicative senescence (30, 31). To test whether CD57 expression can be considered a valid surrogate for cell age, we turned to the UK Adult Twin Register (TwinsUK), a large cohort of monozygotic and dizygotic twins. In a subset of this cohort (*n* = 333), immune phenotypes have been quantified by flow cytometry alongside metadata, including age (32). We found evidence for a positive correlation between an individual’s age and the proportion of CD8^+^CD45RA^+^, CD8^+^CD45RA^–^, CD4^+^CD45RA^+^, and CD4^+^CD45RA^–^ cells that were CD57^+^ (Spearman test *P* = 6 × 10^–9^, 0.06, 4 × 10^–5^, 0.0002, respectively). Although this correlation is between CD57 expression and an individual’s age rather than cell age per se, the two are likely to be linked, and henceforth we used the proportion of cells expressing CD57 as a surrogate of that population’s age.

**Figure 6:**
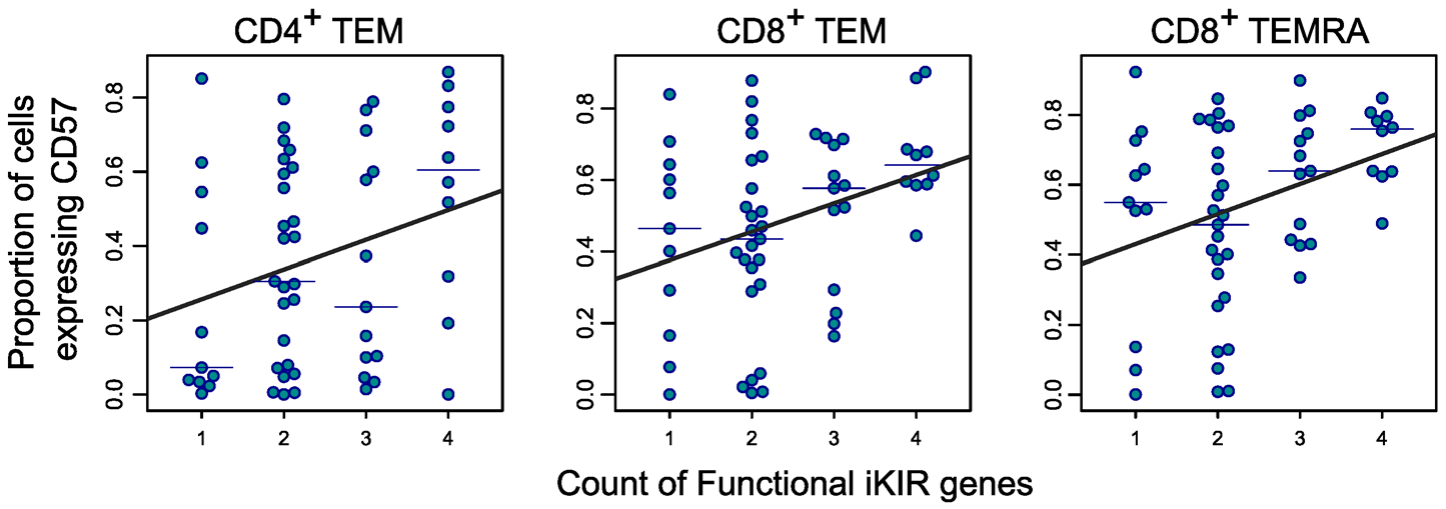
CD57 expression increases with the number of functional iKIR genes carried by an individual. T cell subpopulations with measurable CD57 expression (median ≥150 events) are plotted. The proportion of cells expressing CD57 was significantly positively correlated with the functional iKIR count in CD4^+^ Tem cells (*P* = 0.036), CD8^+^ Tem cells (*P* = 0.018), and CD8^+^ Temra cells (*P* = 0.012). Multivariate regression was done with the age of the individual as a covariate (*n* = 63). Symbols denote experimental measurements, thin horizontal lines the median CD57 expression (for each subpopulation and each functional iKIR count), and thick lines the best fits line of regression.

On this basis, we predicted that in individuals in whom the functional iKIR count is high, the proportion of cells expressing CD57 will be high and that the effect would be most pronounced in older individuals, in whom the effect of the iKIR genotype on cell age would have had the longest time to accumulate. We therefore recruited a cohort of 63 healthy older adults (age ≥60 years, cohort 3) and analyzed the proportion of naive/T stem cell–like memory (naive/Tscm), early Tcm, late Tcm (also called transitional memory), and Tem and Temra CD8^+^ and CD4^+^ cells expressing CD57 by flow cytometry (Supplemental Figure 10). We performed multivariate linear regression with independent variables for the functional iKIR gene count, CMV serostatus, age, and cell differentiation state. We found that for CD8^+^ T cells, CD57 expression was significantly associated with the functional iKIR count (Figure 6, *P* = 0.003), with the percentage of cells expressing high levels of CD57 increasing by 3% (β = 0.03; Supplemental Table 6) for each additional functional iKIR in a person’s genome. To put this into context, the same magnitude of increase in CD57 positivity (3%) was seen with an increase of 12 years in an individual’s age. If naive and Tcm cells (which express very low levels of CD57) were excluded, then the results were even more striking (*P* = 0.0005, 7.8% increase for each additional functional iKIR gene; Supplemental Table 7). For CD4^+^ cells, we observed the same trend, but the effect size was smaller (an increase of 2% for each additional functional iKIR gene) and not significant (*P* = 0.1; Supplemental Table 6). Focusing on the T cell subpopulations with sufficient numbers of CD57^+^ events for accurate analysis (median ≥150 events), namely CD8^+^ Tem, CD8^+^ Temra, and CD4^+^ Tem cells, we found significant associations between CD57 expression and functional iKIR count in each case (*P* = 0.018, *P* = 0.012, *P* = 0.036, respectively; Figure 6). We conclude that our prediction that CD8^+^ T cells will be “older” in people with a high number of functional iKIR genes is consistent with the data. Independent of this interpretation that CD57 expression reflects T cell age, these data show that a functional iKIR gene count affects both CD8^+^ and CD4^+^ T cell immunophenotype.

## Discussion

In this study, we show that the number of iKIR-HLA ligand gene pairs that an individual carries in their genome is a significant predictor of the lifespan of their memory CD8^+^ T cells. In addition to being highly statistically significant (*P* = 3 × 10^–6^), the size of the effect was striking: we found that the CD8^+^ cell lifespan increased by approximately 60 days for each additional functional iKIR gene that a person possessed, resulting in a doubling in T cell survival for a person with 4 functional iKIR genes compared with a person who has 2 functional iKIR genes. Although our previous work had anticipated that iKIR-HLA ligand interactions would increase T cell survival, we still found the size of the effect to be unexpectedly large. There are 2 reasons why such a large effect is striking. First, the variable we are considering, the count of functional iKIR genes, is purely genetic information and considers only 2 gene families (KIR and HLA). A very large number of other factors, both genetic and environmental, would be expected to affect T cell kinetics and to obscure the relationship. The fact that the effect was readily evident despite such variance emphasizes its importance. Second, the genes we consider are, first and foremost, genes that regulate innate immunity; that they have such a marked impact on adaptive T cell survival is remarkable.

A number of pathways by which functional iKIR could enhance CD8^+^ T cell lifespan have been described in the literature. We divided these into “direct” and “indirect” pathways (Figure 1).

The patterns we observed in the data, in particular the strong correlation between functional iKIR count and CD8^+^ T cell lifespan and the absence of any discernible effect of functional iKIR expression on a cell’s lifespan, argue in favor of an indirect mechanism (Figure 5). The direct and indirect mechanisms are not mutually exclusive, and, given the existing data suggesting that KIR expression can directly affect a cell’s survival in vitro and in mice, it is plausible that this also occurs in humans. However, the magnitude of the effect was not discernible in our hands, and we suggest that the indirect pathway, rather than the direct pathway, is the more important determinant of a CD8^+^ T cell’s lifespan in humans in vivo.

We also found that an individual’s functional iKIR gene count had a significant effect on immune aging, specifically on CD57 expression. CD57 expression is often considered a marker of immune aging, although we have previously shown that it is not a marker of replicative senescence (31). In the current study, we showed that CD57 expression was not only significantly positively correlated with functional iKIR count for CD8^+^ T cells but also for CD4^+^ T cells, hinting that functional iKIRs might also impact CD4^+^ T cell survival. Independent of this interpretation, these data point to another important way in which the functional iKIR gene count modulates the immunophenotype. Whether iKIRs modulate other aspects of T cell dynamics such as clonal evolution and diversity is an open question.

We found that iKIR-expressing T cells had higher levels of Ki67 expression than did iKIR^–^ T cells, but, by stable isotope labeling, there was no difference in the proliferation rates of iKIR^+^ and iKIR^–^ cells measured over the longer term. We suggest that Ki67 and iKIRs are transiently upregulated upon activation (consistent with our data as well as previous observations; ref. 29) but that, over a longer time scale (49 days of label administration, approximately 110 days of observation), iKIR expression is not associated with differences in T cell proliferation. That is, Ki67 is better considered as a timestamp of a recently proliferated cell (33). This serves as a caveat against the interpretation of Ki67 as a measure of in vivo lifespan and emphasizes the importance of direct in vivo measurements such as those made using stable isotopes. This conclusion is echoed by animal studies that also showed substantial differences between Ki67 expression and more direct measures of cell proliferation (34).

We suggest that, together, these data provide a mechanistic explanation for our previous observations that an increased number of iKIR-HLA ligand gene pairs significantly enhances CD8^+^ T cell–mediated control of virus in HIV-1, HCV, and HTLV-1 infections. Mathematical modeling showed that these observations could be explained if iKIR-ligand interactions increased the survival of CD8^+^ T cells (8). Furthermore, in a longitudinal study of an HIV-1–infected cohort, this previous work also showed that protective HLA-B*57 and detrimental HLA-B*35Px associations were both better maintained over time in people with a high number of functional iKIR genes compared with people with a low number, again consistent with the hypothesis that the enhancement of HLA associations observed is related to increased T cell survival. Very recently, we have found that the number of functional iKIR genes also affects HLA associations with the risk of type 1 diabetes (our unpublished observations), suggesting that the process we have identified in the context of chronic viral infection is also relevant in some cases of autoimmunity. Although it is very early to be speculating about therapeutic implications, 2 facts are pertinent: first, that this pathway is clinically relevant and second that this pathway is druggable. Monoclonal antibodies (mAbs) that are capable of blocking or activating iKIRs in vitro have been developed. Lirulimab, a mAb that blocks KIR2DL1 and KIR2DL2/L3, has been developed as an NK therapeutic and tested in human clinical trials to treat solid tumors and hematologic malignancies (35); it could potentially be repositioned to alter T cell lifespan. Some indirect mechanisms, e.g., an impact of iKIRs on NK cell education may be harder to drug, as the effects may have already occurred during development. Nevertheless, the requirement for NK signaling must still be met for effector function and can potentially still be targeted. In the long term, there is, therefore, the possibility to modulate T cell responses in vivo by dampening (by KIR blocking) or boosting (by KIR ligation) T cell survival depending on the context.

Another potentially important direction for translation is in allo-hematopoietic stem cell transplant donor-recipient matching. Several studies have already considered the effect of donor KIR genotype and donor-recipient KIR ligand mismatches on NK cell alloreactivity (36–38). Our work suggests that donor NK cell autoreactivity may also be relevant. An essential prerequisite for translation is a detailed understanding of the underlying mechanism. For this, an animal model would be invaluable. The extent to which murine Ly49 receptors adequately model the human iKIR is unclear. One step would be to see whether the relationship we observed between the count of functional iKIR genes and CD8^+^ cell lifespan in humans is recapitulated by murine models.

One limitation of this study is that the count of functional iKIR genes that we used was relatively simple and did not incorporate iKIR allele–level information (other than the broad allotypes of KIR2DL2/L3 and KIR3DL1/S1). There are 2 main reasons for the decision. First, our aim was to investigate possible mechanisms underlying our prior observation that the functional iKIR count enhanced HLA class I associations with clinical outcomes, and so we needed to use this previous definition of the functional iKIR count (which did not include allele-level information) for comparability. Second, it is far from clear how allele-level information should be incorporated into the functional count: Should some alleles count as less than 1? If so, which alleles and what count should they be assigned? Should the “count” of a KIR-HLA allele pair only reflect its strength of signaling, or should other factors such as the expression level of KIR and HLA also be incorporated? Our first attempt at incorporating some of these details (the weighted inhibitory score) was also a significant predictor of CD8^+^ T cell lifespan but it did not perform better than the unweighted count of functional iKIR genes. In stark contrast, a count of the number of iKIR genes, without taking into account whether they were functional, performed very poorly, as we expected, suggesting that, though simple, there is content in our definition of a functional iKIR gene. Ultimately, a much larger study would be needed to interrogate such subtleties in the future.

To summarize, the data presented here show that the iKIR-HLA ligand genotype has a profound impact on CD8^+^ T cell lifespan as well as on CD4^+^ and CD8^+^ T cell immunophenotype. Furthermore, since these are relationships between genotype and phenotypes, the direction of causality is unequivocal. Separately, we have also shown that the iKIR-ligand genotype has direct and measurable consequences for human health, as it affects the risk of developing type 1 diabetes, the rate of progression to a low CD4^+^ count in people living with HIV-1, the odds of spontaneous clearance of HCV, and the risk of developing inflammatory disease in the context of HTLV-1 infection. The wide range of diseases involved and the central role of T cells in human immunity suggest that this iKIR impact on CD8^+^ T cell lifespan may be of fundamental importance.

## Methods

### Experimental data

#### Study participants.

A total of 134 participants across 3 cohorts were recruited. Cohort 1 (KIR expression analysis and stable isotope labeling) comprised 23 healthy adults including uninfected individuals serving as controls (*n* = 4); individuals with viremic, untreated HCV (*n* = 9); individuals with aviremic, treated HIV-1 (*n* = 7); and individuals with untreated HTLV-1 (*n* = 3). Individuals with no functional iKIRs were excluded from the study (as we needed some functional iKIR expression to investigate the direct hypothesis). For the labeling analysis, the individuals carrying a KIR3DL2 ligand were initially excluded (see below), leaving 18 individuals.

Cohort 2 (KIR expression analysis — replication cohort) comprised 48 healthy adults, including uninfected individuals serving as controls (*n* = 33) and individuals with viremic, untreated HCV (*n* = 15). Hepatitis C viral loads ranged from 2.4 × 10^4^ to 3.3 × 10^6^, with a median of 1.8 × 10^6^ RNA copies/mL. All individuals in cohort 2 were seronegative for HIV-1 infection.

Cohort 3 (CD57 expression analysis) comprised 63 healthy older adults (≥60 years of age; range, 60–91 years; median, 75 years), all of whom were seronegative for HIV-1, hepatitis B, and HCV infection.

#### KIR expression analysis.

For analysis of cohort 1, CD4^+^ and CD8^+^ T cells were gated into 4 subpopulations: naive/Tscm (CD45RA^+^CD28^+^), Tcm (CD45RA^–^CD28^+^), Tem (CD45RA^–^CD28^–^), and Temra (CD45RA^+^CD28^–^) (see Supplemental Methods for panel details including clone names and Supplemental Figure 1A for representative gating). iKIR antibodies specific for KIR2DL1, KIR2DL2/L3, and KIR3DL1 were conjugated to different dyes, enabling analysis of coexpression, and cells were analyzed and sorted on a BD FACSAria III (BD Biosciences). Cytometric data were quantitated using FlowJo.

For analysis of cohort 2, T cell subpopulations were defined as above. Phycoerythrin-conjugated (PE-conjugated) iKIR-specific antibodies were used to stain samples separately for each iKIR studied. Cells were analyzed on a BD FACSCanto II cytometer (BD Biosciences) and data were quantified using FlowJo.

#### CD57 expression analysis.

CD4^+^ and CD8^+^ T cells were gated as described above. To permit higher resolution, Tcm cells were further separated into early Tcm and late Tcm cells (also called transitional memory [Ttm]) (39) by CCR7 staining. For enumeration of highly differentiated cells, staining with CD57 was performed (see Supplemental Figure 10 for representative gating).

#### Stable isotope labeling in vivo.

We have previously described the isotope labeling protocol in detail (40). Briefly, participants were given oral doses of 70% ^2^H_2_O over a 7-week period (50 mL 3 times daily for 1 week, then twice daily thereafter). Saliva samples were collected for evaluation of body water labeling. Peripheral blood was collected at successive time points during and after labeling, and PBMCs were separated by Ficoll gradient centrifugation. For normalization, monocytes, a cell population expected to reach fully labeled status during the labeling phase, were sorted from an aliquot of PBMCs by CD14 magnetic bead column positive selection (MACS, Miltenyi Biotec). A BD FACSAria III flow cytometer was used to sort PBMCs into CD8^+^ Tcm and CD8^+^ Temra cells on the basis of cell-surface expression of canonical differentiation markers (Tcm: CD45RA^–^CD28^+^; Temra: CD45RA^+^CD28^–^). Both subsets were further sorted on the basis of their iKIR expression into cells (Tcm or Temra) expressing functional iKIRs and cells expressing nonfunctional iKIRs (if the latter was not available or cell numbers were too low, then cells that were iKIR^–^ were collected instead). Cells from each individual had a bespoke gating strategy based on their HLA and KIR genotype, as each individual had different functional and nonfunctional iKIRs. Given the low or absent cell frequencies, it was not possible to collect all cell populations for all individuals (see Supplemental Figure 1, A and B for representative gating). Deuterium enrichment in the DNA of monocytes and sorted T cell subpopulations was measured by gas chromatography/mass spectrometry of the pentafluorobenzyl derivative as previously described (23, 41).

#### Definition of functional and nonfunctional iKIR expression.

We used the following rules to define cells expressing functional or nonfunctional iKIRs. A cell was classified as expressing functional iKIRs if it expressed KIR2DL1, KIR2DL2/L3, and/or KIR3DL1, and the individual was positive for any HLA allele encoding a corresponding ligand (KIR2DL1: *C2*; KIR2DL2: *C1* [which includes HLA-B*46 and -B*73], *C2*; KIR2DL3: *C1* [including HLA-B*46 and -B*73]; KIR3DL1: *Bw4* [which includes HLA-A*23, -A*24, and -A*32, together with some other rare HLA-A alleles], using the definitions of iKIR ligands provided in the Immuno Polymorphism Database; refs. 4, 42). A cell was classified as expressing nonfunctional iKIRs if it both (a) expressed an iKIR (KIR2DL1, KIR2DL2/L3, and/or KIR3DL1) for which the individual did not carry an allele encoding any corresponding HLA ligand and (b) did not express any functional iKIR (i.e., we assumed that the signal for a functional iKIR was dominant, and this cell would instead be classified as expressing functional iKIRs).

In the case in which KIR2DL2 was functional and KIR2DL3 was nonfunctional, then cells expressing KIR2DL2/L3 could not be unambiguously classified (as either functional or nonfunctional) and thus were not collected.

There is evidence that KIR3DL2, a framework KIR, behaves differently from the other iKIRs, particularly its exceptionally strong binding to HLA-B*27 heavy-chain homodimers and non-HLA ligands (2, 26–28, 43, 44), and we were concerned it may confound our results. Therefore, for donors negative for the alleles encoding HLA ligands of KIR3DL2 (HLA-A*03, A*11, B*27), those cells expressing KIR3DL2 were excluded from the sorted populations (directed into the dump channel), whereas cells from donors that were positive for any of the alleles encoding HLA ligands of KIR3DL2 (*n* = 5) were removed from the analysis in the first instance, reducing the cohort size from 23 to 18. For the sensitivity analysis including those individuals positive for the classical KIR3DL2 ligands HLA-A*03 and -A*11 (*n* = 2), the cells negative for KIR3DL2 were included and classified according to the standard definitions (nonfunctional or iKIR^–^); cells from other individuals kept their original classifications.

#### In vitro activation studies.

PBMCs from 10 healthy donors were either cultured in media alone or stimulated with purified anti–human plate-bound CD3 and soluble CD28 (2 μg/mL and 1 μg/mL, respectively, BD Biosciences). Expression of iKIRs and CD38 on CD8^+^ T cells was measured by flow cytometry at 6, 12, and 24 hours. For iKIR expression, a cocktail of PE-conjugated KIR2DL1-, KIR2DL2/L3-, and KIR3DL1-specific antibodies was used. To assess the stability of iKIR expression, PBMCs were stimulated as above, sorted into iKIR^+^ and iKIR^–^ fractions, and allowed to rest for 72 hours. iKIR expression was evaluated by flow cytometry at the time of sorting (0 h) and at 72 hours.

#### Genotyping.

High-resolution HLA typing for the HLA-A, B, and C loci was carried out by the Antony Nolan Trust using next-generation sequencing. KIR genotyping of cohort 1 was performed by the qKAT multiplex qPCR method using a Roche LightCycler 480 (45); KIR genotyping of cohort 2 was performed by PCR-SSP following the method of Vilches et al. (46); and KIR genotyping of cohort 3 was performed by high-throughput sequencing (47) and the results interpreted using the bioinformatics pipeline PING (48).

### Mathematical modeling

#### Fitting the stable isotope–labeling data.

There are 3 parts in the model to estimate the proliferation and disappearance rate of the T cell subsets. First, we quantified the availability of label in body water by measuring the fraction of heavy water in the saliva. We describe this availability with an empirical function *S(t)* with 3 parts (Equation 1) to reflect the 3-part protocol (full dose for 7 days, 2/3 dose for 42 days, delabel) (Supplemental Figure 3). Individuals were assumed to have different values of *f* (the maximal fraction of heavy water in the body, attained asymptotically), but δ (the rate of turnover of body water per day) was constrained to be the same for everyone (since the turnover of body water is expected to be similar among individuals). Replacing the empirical function with a piece-wise function gave very similar results.

 (Equation 1) 
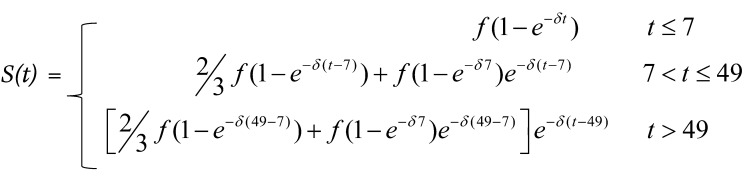


Next, for each individual, we modeled the fraction of label in a rapidly-turning-over cell population (monocytes) in order to estimate the amplification factor *b_w_* (also referred to as *c*; ref. 21); this is a factor that reflects the increase in M+1 when a cell divides, given enrichment *S(t)*, i.e., it scales between label enrichment in newly synthesized DNA and precursor availability in body water (17). We describe the label enrichment in DNA of monocytes (Equation 2) using a previously proposed mechanistic model (19, 49):

 (Equation 2) 
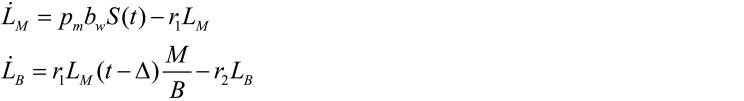


In Equation 2, *L_M_* is the fraction of label in bone marrow monocyte precursors, *L_B_* is the fraction of label in blood monocytes (the observable), *p_m_* is the proliferation rate of precursors, *r_1_* is the rate of exit from the mitotic pool in bone marrow, Δ is the time spent in the postmitotic pool in bone marrow, *M/B* is the ratio of the number of monocytes in the bone marrow to the number of monocytes in the blood, *S(t)* is the saliva enrichment estimated for that individual in step 1, and *b_w_* is the amplification factor of interest (see Supplemental Figure 4). We used equilibrium constraints to eliminate *p_m_* and *r_1_*. *M/B* and Δ were fixed at estimates of 2.6 days and 1.6 days, respectively (19, 49) (however, we showed that the estimates of *b_w_* were independent of these values; this follows because *b_w_* depends only on the plateau enrichment in blood monocytes). Thus, there were 2 free parameters (*b_w_* and *r_2_*) that were estimated by fitting the model to the data; different values were allowed for each individual, and we used uniform priors of [0,7] and [0,10] for *b_w_* and *r_2_*, respectively. Finally, we used the information from steps 1 and 2 to describe the label enrichment in T cells. The equations for the fraction of label in T cells are as follows:

 (Equation 3) 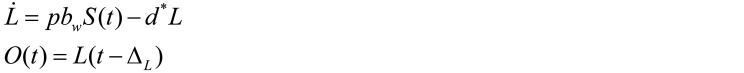


In Equation 3, *L* is the fraction of label in the DNA of the T cell subset at the site of division, *p* is the proliferation rate of T cells, *S(t)* is the saliva enrichment estimated in step 1 above, *b_w_* is the amplification factor estimated in step 2 above, d* is the disappearance rate of labeled T cells, *O(t)* is the observed label at time *t*, which is the label at the site of division (likely lymphoid tissue) lagged by a time Δ*_L_* to reflect a delay of Δ*_L_* days for a T cell to traffic from the site of division to the blood (where it is observed). The parameters *p*, d*, and Δ*_L_* are drawn, for each T cell subset, from a lognormal prior distribution whose parameters are fitted (i.e., a hierarchical model). The lognormal distribution has 2 parameters: μ and σ. The prior for σ*_p_*, σ_d*_, and σ_Δ*L*_ is uniform between 0 and 1. The priors for μ*_p_*, μ_d*_, and μ_Δ*L*_ are uniform between – ∞ and log(0.05), log(1), and log(21), respectively. Using a hierarchical model improved convergence compared with allowing parameters to vary freely. Note that the hyperparameter μ*_p_* is the same for all lymphocyte populations, regardless of infection status, functional iKIR count, cell subpopulation (CD8^+^ Tcm or Temra), and iKIR expression status (functional, nonfunctional, iKIR^–^); i.e, at no point did we impose any assumptions on how *p* varied between the cell populations. Equation 3 was solved analytically to speed up the fitting process. All model fitting was conducted within a Bayesian framework, using the NUTS sampler implemented in Stan (via the R package rstan) (50). The saliva, monocyte, and lymphocyte data were fitted simultaneously across all individuals and lymphocyte subsets. Simultaneous fitting of saliva, monocyte, and lymphocyte data means that errors are propagated correctly (fitting in a stepwise manner, as is common [ref. 21], with point estimates from the first step being used in the second step and so on will lead to an underestimate of errors on the final parameters). To summarize, the parameters *f*, Δ, *b_w_*, *r_2_*, *p*, d*, and Δ*_L_* were fitted; *f*, *b_w_*, and *r_2_* were allowed to be different for each individual; *p*, d*, and Δ*_L_* were allowed to be different for each individual and each cell population; Δ was constrained to be the same for all individuals; and the total number of data points was 1,085 (not including replicates, typically 3 per data point). Best fits of the model to the saliva and monocyte data are shown in Supplemental Figures 3 and 5, respectively. Best fits of the model to the T cell data are shown in Figure 3 and Figure 4. Parameter estimates are provided in Supplemental Tables 3 and 4. The model fitting code is available at https://github.com/ada-w-yan/kirdynamics (commit ID: e99899b). Repeating the fits in a frequentist framework using the global optimizer pseudo from the package FME (51) in R, version 4.1.2, gave virtually identical results. The cell lifespan we report is the average for each subpopulation: it is defined as *1/p* and is the average time between the creation of a cell (by proliferation) and the loss of the cell (by proliferation, death, differentiation); it differs from half-life by a factor of ln(2), i.e., half-life = *ln(2).lifespan=ln(2)/p*.

#### Model prediction of the relationship between cell survival and cell age.

We calculated how a change in the lifetime of a memory CD8^+^ T cell would impact CD8^+^ T cell age. We modeled the turnover of CD8^+^ T cells as follows:

 (Equation 4) 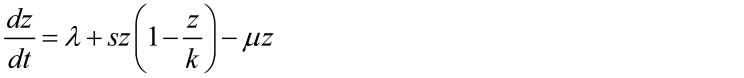


In Equation 4, *z* is the concentration of CD8^+^ T cells, λ is the rate at which cells enter the memory T cell compartment, *s* is the rate of division, *k* is the carrying capacity of CD8^+^ T cells, and μ is the death rate.

The lifetime of CD8^+^ T cells in this model, as measured by stable isotope labeling, is 1/μ. Lifetime is defined as the time from cell production, either by division or by entry to the memory compartment, to its death.

We defined cell age as the time (in days) since the cell or its ancestor entered the memory compartment, not the time since the last division. If we define *w*(*a*, *t*) as the concentration of CD8^+^ T cells of age *a* at time *t*, then:

 (Equation 5) 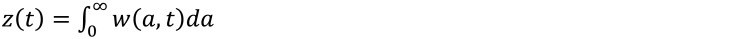


The equation for *w*(*a*, *t*), derived from the von Foerster equation (52), is as follows:

 (Equation 6) 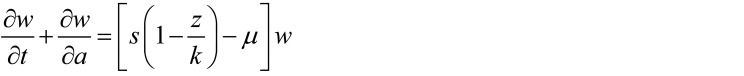


In Equation 6, the initial condition is *w*(*a*, 0) = *f*(*a*), where *f*(*a*) is the initial age distribution, and the boundary condition is *w*(0,*t*) = *λ*.

We solve the steady-state age distribution by letting *z* = *¯z*, where bars denote the steady-state value. As *¯z* is a constant, the age equation is of the same form as if there were no proliferation, and a constant death rate equal to *D* = *μ* – *s* (1 – *¯z*/*k*). Therefore, the age distribution at equilibrium is exponential with rate parameter *D*, and the mean age is 1/*D*.

We can show (Supplemental Results 5) that *dD/dμ* > 0 for all positive values of model parameters. Therefore, CD8^+^ T cell age increases as the CD8^+^ T cell lifetime increases, all other parameters being equal. However, the rate parameter *D* is the difference of 2 similar terms, both of which might be expected to vary with the functional iKIR count, and thus, while the intuition that cell age would increase with the functional iKIR count due to an increase in cell survival is correct, the correlation is expected to be weak.

### Statistics

All statistical analysis was carried out in R, version 4.1.2. All reported *P* values are 2 tailed. A *P* value of less than 0.05 was considered significant, and where this threshold was decreased because of testing of multiple hypotheses, it is noted in the text. Multivariate linear regression, ANOVA, Davidson-MacKinnon J tests, and paired Mann-Whitney *U* tests (Wilcoxon) were conducted with the functions lm, anova, lmtest::jtest, and stats::wilcox.test, respectively (base package unless stated otherwise).

### Study approval

All study procedures were conducted according to the principles of the Declaration of Helsinki. All participants gave written informed consent following protocols approved by the National Research Ethics Service (NRES) (London 13/LO/1621 and 13/LO/0022, South Central Oxford 15/SC/0089/2).

## Author contributions

LB analyzed the data and contributed to the study design. LH recruited the participants and conducted experiments. AS, YZ, and DPG conducted experiments. LS, LMB, and AWCY analyzed the data. NNG, DK, and CR conducted the experiments. JAT supervised the experiments. GPT recruited the participants and provided patient data. DF recruited the participants. PJN and SGEM supervised the experimental work. RB supervised the experimental work and analysis and contributed to the study design and conception. DCM supervised the experimental work, contributed to the study design, and wrote the manuscript. BA analyzed the data, supervised the data analysis, conceptualized and designed the study, acquired funding, and wrote the manuscript. The authorship order of the co–first authors is based on the relative contributions of their input to the final manuscript.

## Supplementary Material

Supplemental data

ICMJE disclosure forms

## Version 1 04/18/2023

In-Press Preview

## Version 2 06/15/2023

Electronic publication
