# Supplementary material for: KIR-HLA interactions extend human CD8+ T cell lifespan in vivo
Source: J Clin Invest. 2023 Jun 15;133(12):e169496. doi: 10.1172/JCI169496 (PMC10266773; doi:10.1172/JCI169496)
Supplement: Supplemental data [file jci-133-169496-s276.pdf]

Supplementary Material Contents

Supp pg 1 ….. Supplementary Figures 1-10

Supp pg 12 ….. Supplementary Tables 1-7

Supp pg 20 ….. Supplementary Methods

Supp pg 22 ….. Supplementary Results 1-5

Supp pg 24 ….. Supplementary References

Supplementary Figures

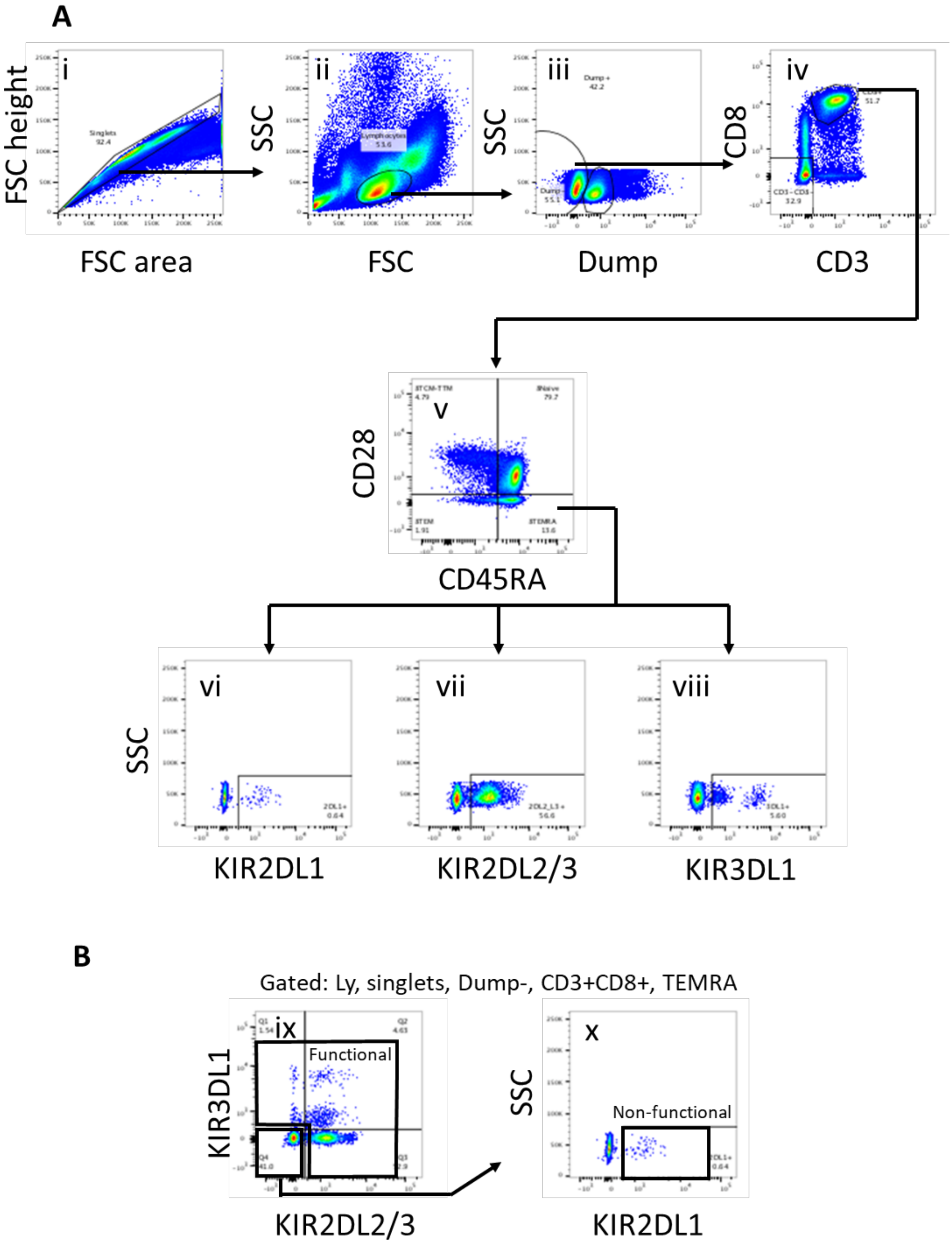

Supp Figure 1. Gating strategy for enumeration and sorting of iKIR^{+} CD8^{+} memory T subsets for volunteers in Cohort 1.

The example shows an analysis of PBMCs from a healthy donor (DW14, obtained at week 5 of $^{2}H_2O$ labelling). The frequencies of relevant cell populations were stable throughout the sampling period. **(A)** For analysis, serial gating was used to identify (i) single cells based on forward scatter (FSC) pulse height and area; (ii) intact lymphocytes based on forward and side

scatter; (iii) cells negative in the dump channel (excluding unwanted lineages [CD4, CD14, CD19] and necrotic cells using a live/dead discriminator dye); and (iv) $CD3^+CD8^+$ T cells. (v) Within the CD8+ T cells, CD45RA and CD28 staining was used to distinguish populations of naïve/T_{SCM} cells ($CD45RA^+CD28^+$), T_{CM} cells ($CD45RA^-CD28^+$), T_{EM} ($CD45RA^-CD28^-$), and T_{EMRA} ($CD45RA^+CD28^-$). Each of these four subsets was gated (T_{EMRA} are shown as an example) to determine positive staining for (vi) KIR2DL1; (vii) KIR2DL2/KIR3DL3 (which cannot be readily distinguished by available mAbs); or (viii) KIR3DL1. Boundaries for positive staining in (vi-viii) were determined using FMO controls.

(B) For sorting of the $CD8^+$ T cells, individual HLA genotypes were used to determine whether an iKIR was "functional" (one or more alleles encoding the HLA ligand carried by the donor) or "non-functional" (HLA ligand absent). The donor in this example carried the three iKIR genes KIR2DL1, KIR2DL2/L3 and KIR3DL1; they also carried HLA alleles with the C1 and Bw4 motifs but not the C2 motif. Therefore, KIR2DL2/3 and KIR3DL1 were functional and KIR2DL1 was not. This information was then used to design a bespoke gating strategy for this individual. $CD8^+$ T_{CM} (not shown) or T_{EMRA} were gated as in (i-v), except that the lymphocyte scatter gate was applied before the doublet discriminator and KIR3DL2-expressing cells were directed to the dump channel. (ix) Functional iKIR were sorted as cells expressing KIR2DL2/3 or KIR3DL1 or both. (x) We then sorted cells that expressed non functional iKIR (in this case KIR2DL1 but not KIR2DL2/L3 or KIR3DL1), having gated on cells lacking functional iKIR expression (bottom left in (ix)). In some donors in whom the frequency of non-functional iKIR was too low for adequate sort yields, cells that expressed no iKIRs were sorted as an alternative population.

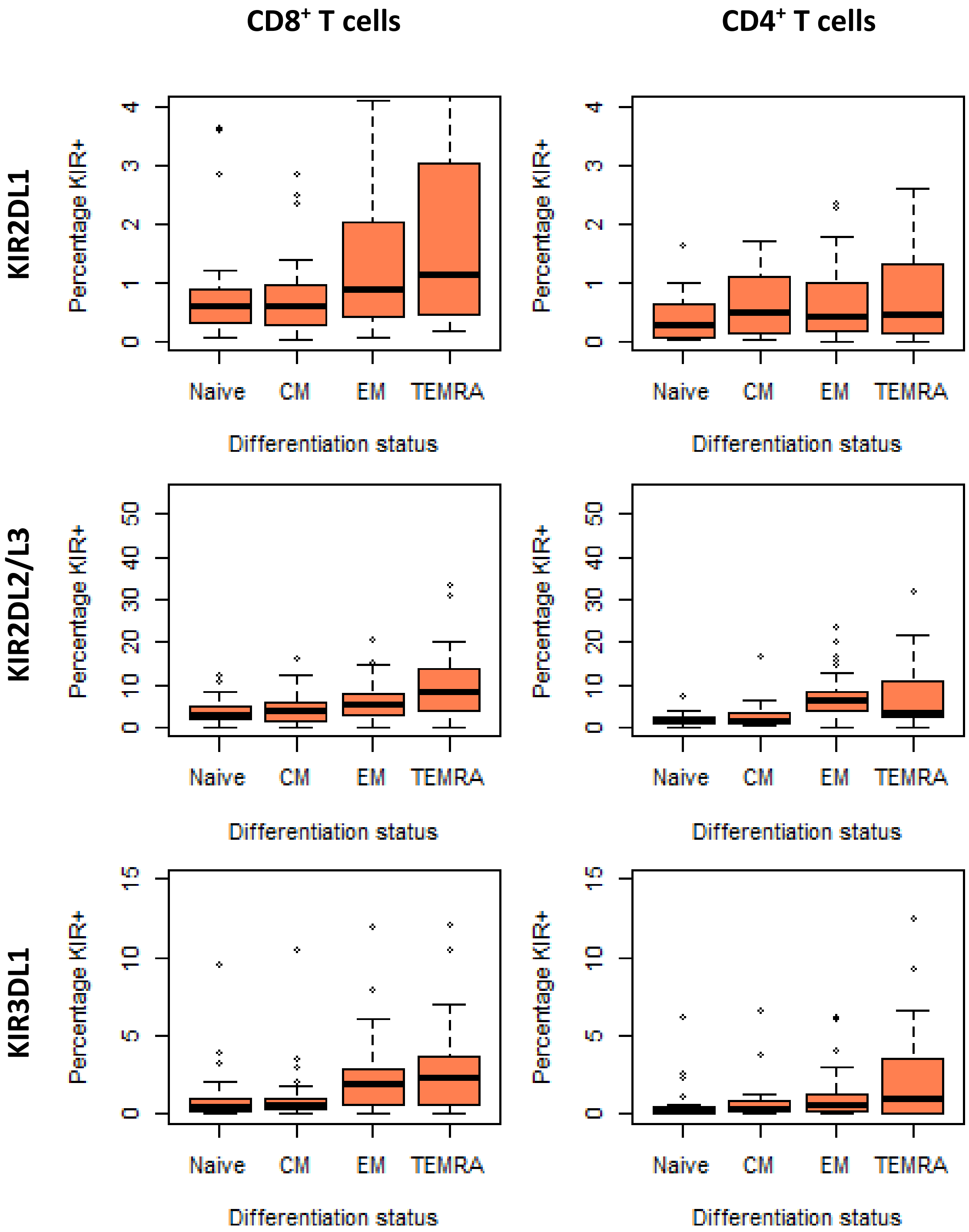

Supp Figure 2. Percentage of T cells expressing different inhibitory KIR in cohort 2. The percentage of cells in each T cell subpopulation expressing 3 different iKIR was quantified by flow cytometry. Note the scale of the y axis is different between the rows. Boxes show medians and interquartile ranges.

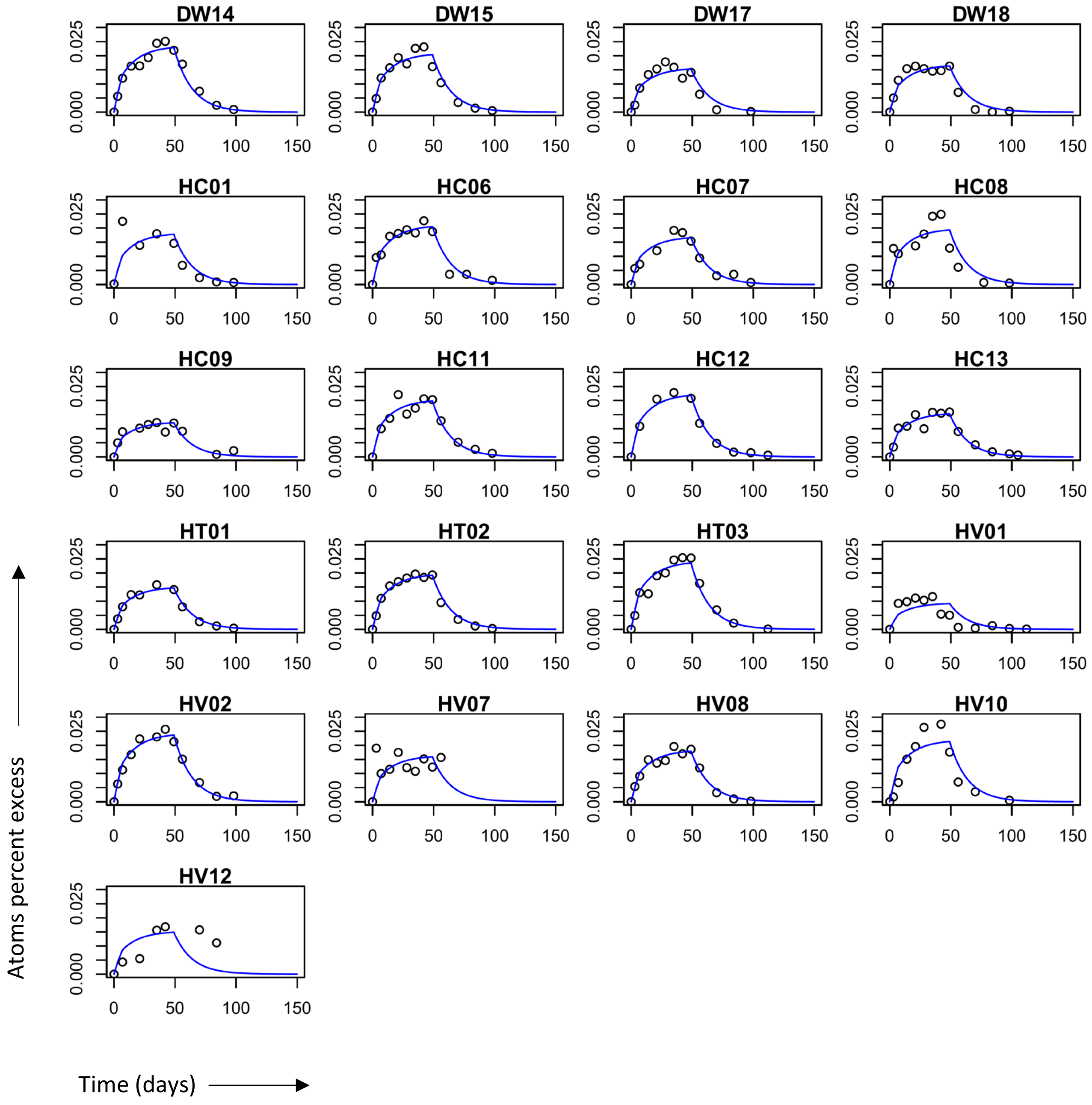

Supp Figure 3. Label enrichment in saliva. Plots show, for each individual, the measured deuterium enrichment in the water component of saliva (circles) and the best fit of the empirical function to the data (blue line). Two individuals carrying HLA-B*27 (which binds as a heavy chain homodimer abnormally strongly to KIR3DL2) were excluded.

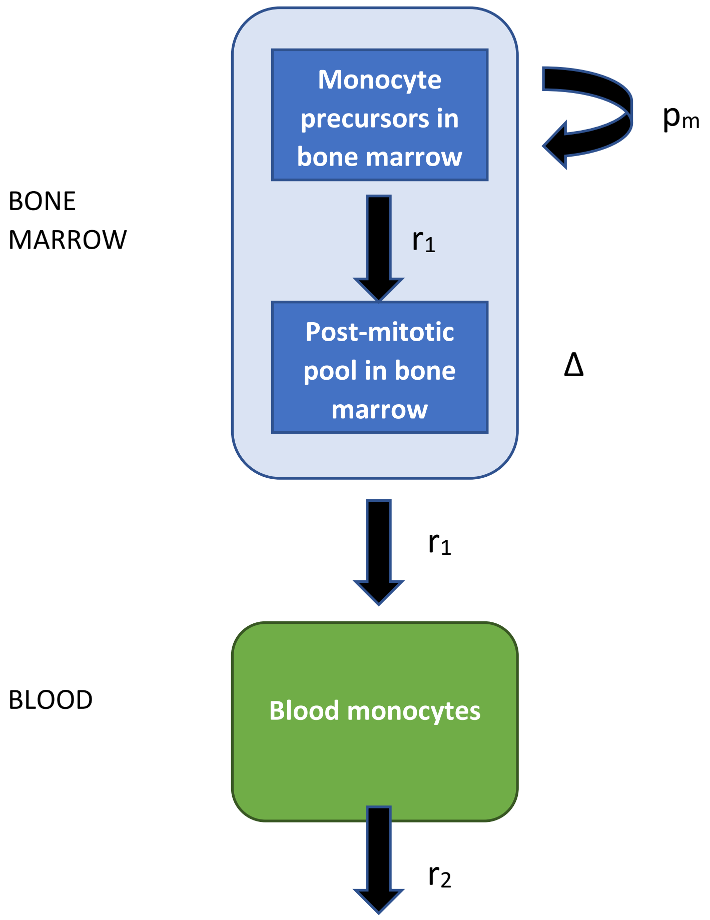

Supp Figure 4. Schematic of Mechanistic Model to Describe Monocyte Kinetics. Here p_m is the proliferation rate of precursors, r_1 is the rate of exit from the mitotic pool in bone marrow, Δ is the time spent in the post-mitotic pool in bone marrow and r_2 is the rate of disappearance of blood monocytes (death and long-term exit from the blood).

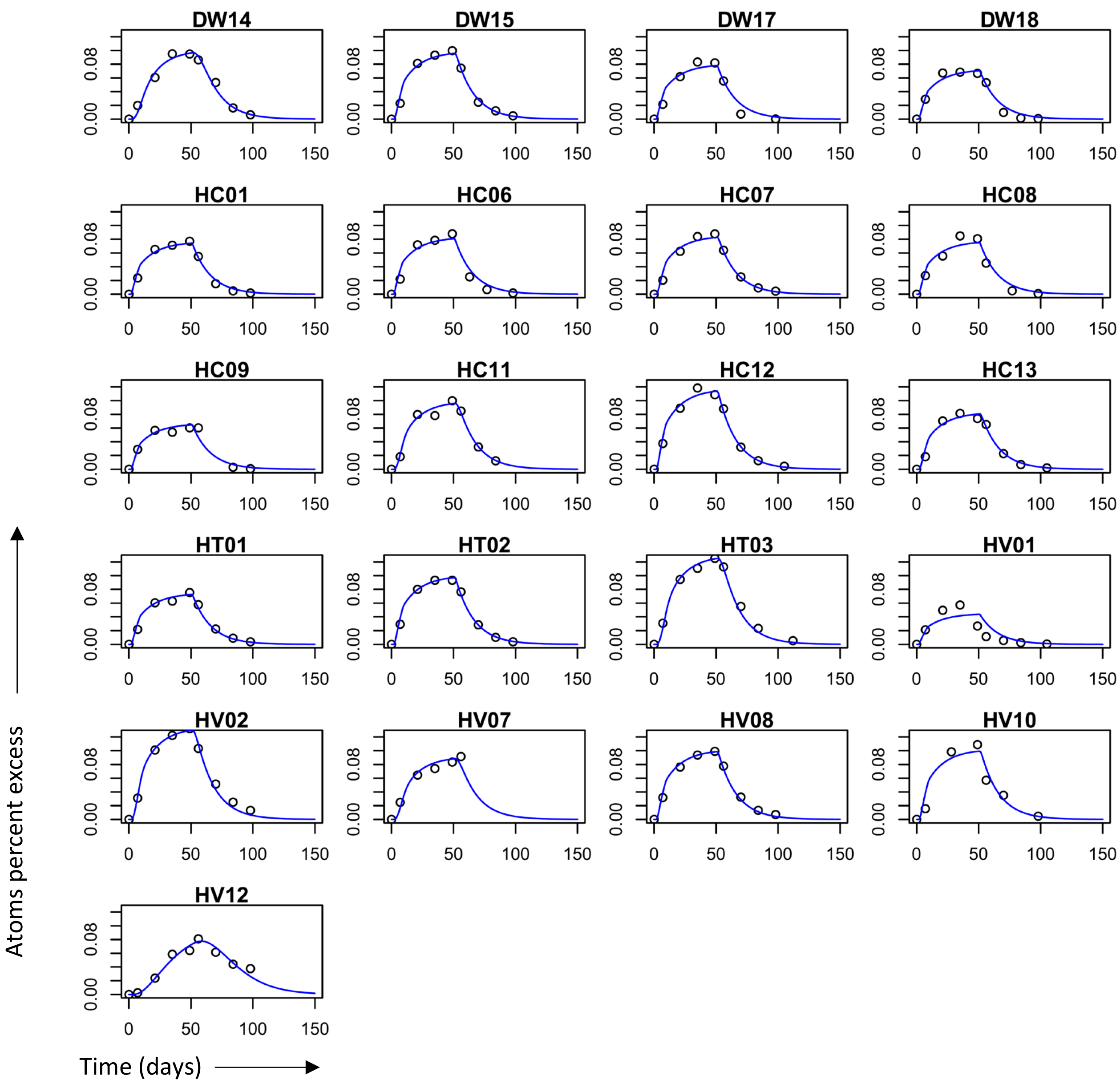

Supp Figure 5. Label enrichment in monocytes. Plots show, for each individual, the measured deuterium enrichment in DNA extracted from monocytes (circles) and the best fit of the mechanistic model to the data (blue line). Two individuals carrying HLA-B*27 (which binds as a heavy chain homodimer abnormally strongly to KIR3DL2) were excluded.

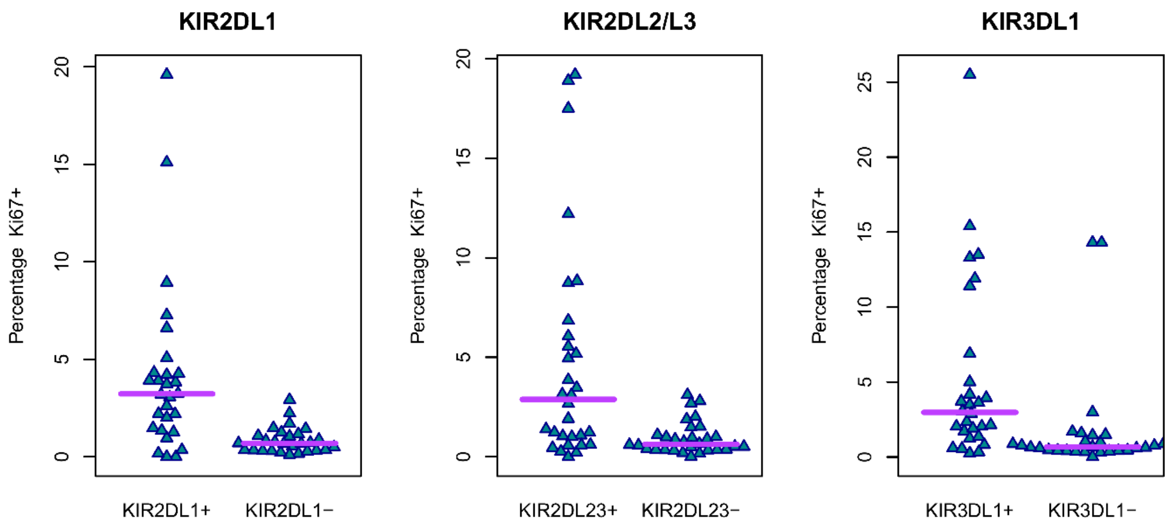

Supp Figure 6. Percentage of iKIR$^+$ and iKIR$^-$ CD8$^+$ T cells expressing Ki67 in Cohort 2. The percentage of KIR$^+$ and KIR$^-$ cells expressing Ki67 was quantified by flow cytometry for each of three different iKIR: KIR2DL1, KIR2DL2/L3, KIR3DL1 in turn. In each case, Ki67 expression was significantly higher in the KIR$^+$ population than in the KIR$^-$ population (P=1x10^{-7}, 1x10^{-7}, 6x10^{-4}; Wilcoxon signed rank test paired two tailed). This relationship was independent of whether the iKIR was functional or not.

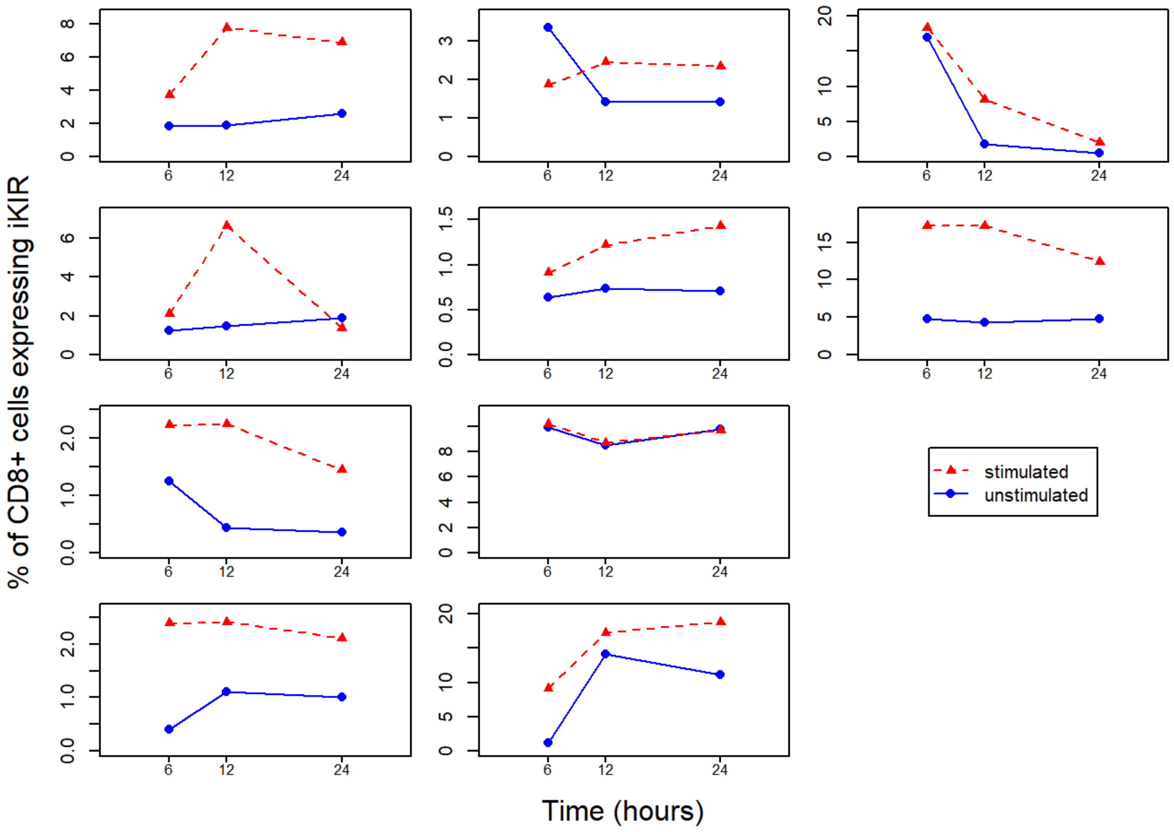

Supp Figure 7. iKIR expression is upregulated on T cells upon stimulation. PBMC from 10 donors were split into two conditions and either cultured in media alone or with αCD3αCD28. iKIR expression on $CD8^+$ cells was measured by flow cytometry at 6, 12 and 24h. In the graph above, one donor is shown in each panel, the red dashed lines with triangles depict iKIR expression (KIR2DL1, KIR2DL2/L3 and/or KIR3DL1) in the stimulated condition and blue solid lines with circles depict iKIR expression in media only condition. iKIR expression was significantly increased by stimulation at 6h (P= 0.027, Wilcoxon signed rank test); the same pattern is seen at 12h (P= 0.002) and 24h (P= 0.01).

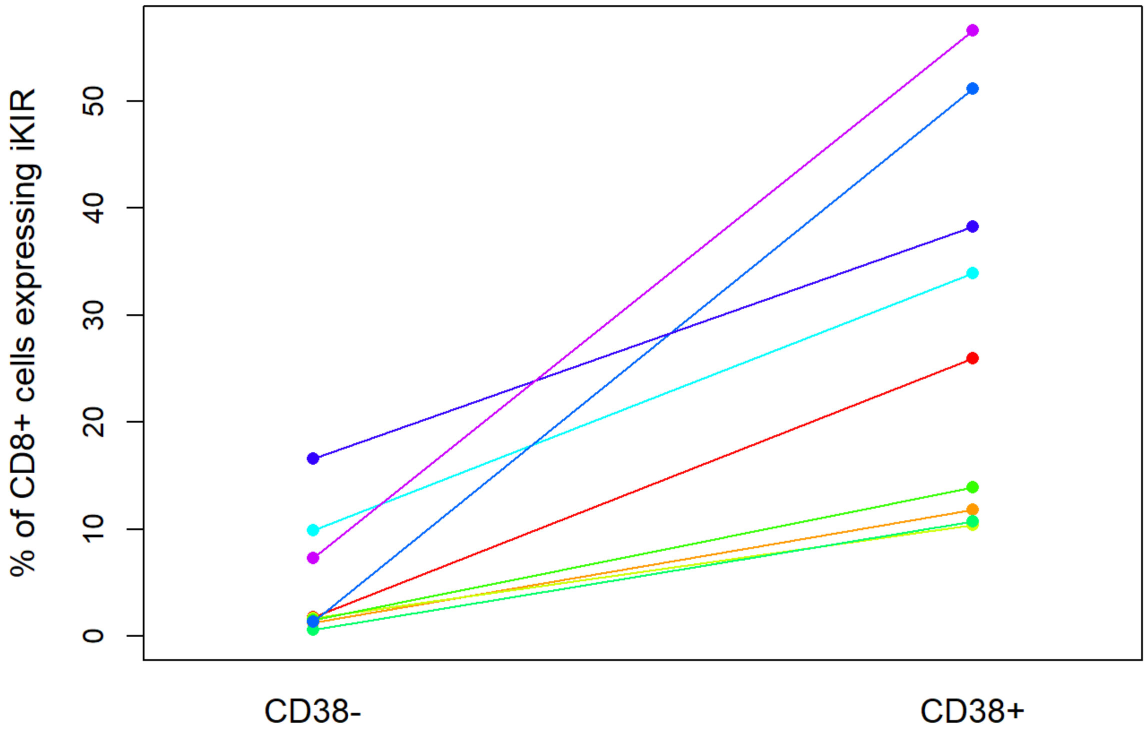

Supp Figure 8. iKIR expression is elevated on activated CD8$^+$ cells in vitro. PBMC from 10 donors were cultured with αCD3αCD28. iKIR expression (KIR2DL1, KIR2DL2/L3 and/or KIR3DL1) on quiescent (CD38$^-$) and activated (CD38$^+$) CD8$^+$ cells was measured by flow cytometry at 6h. iKIR expression was significantly higher on CD38$^+$ cells than on CD38$^-$cells (P=0.004, Wilcoxon signed rank test). One line per donor.

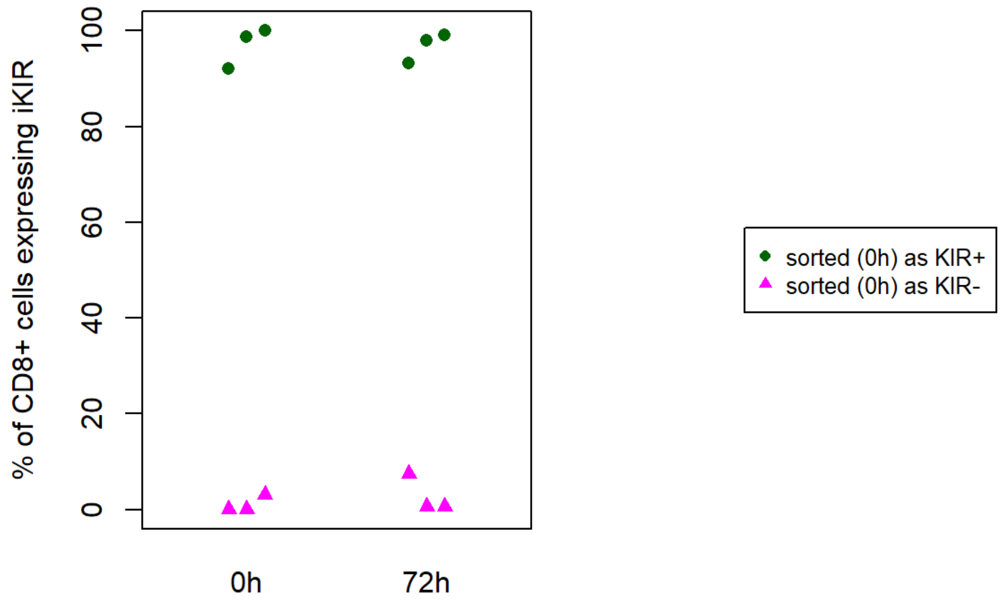

Supp Figure 9. iKIR expression is stable at a cellular level for 72h. PBMC from 3 donors were stimulated with αCD3αCD28. $CD8^{+}iKIR^{+}$ and $CD8^{+}iKIR^{-}$ lymphocytes were sorted, resuspended and allowed to rest for 72h. iKIR expression was quantified at 0h (upon sorting) and at 72h. Green circles denote iKIR expression on cells sorted (0h) as $iKIR^{+}$, pink triangles denote iKIR expression on cells sorted (0h) as $iKIR^{-}$. We find that iKIR expression (and the lack of it) is stable at a cellular level for 72h.

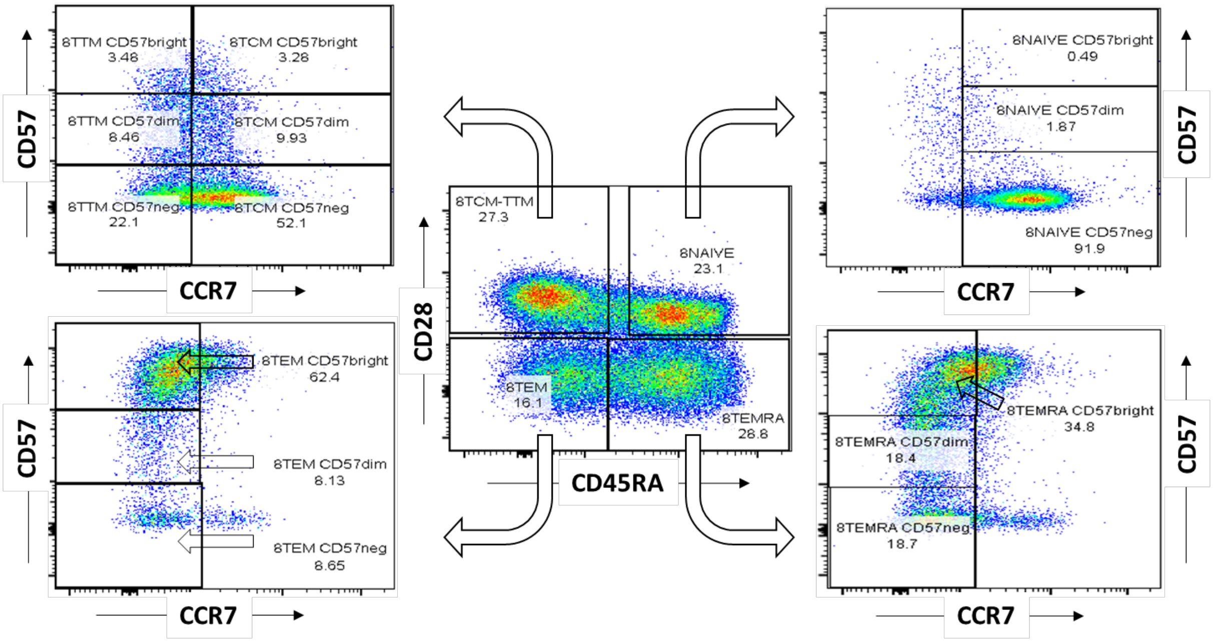

Supp Figure 10. Representative gating for analysing CD57 expression. Cell gates representing the Naïve/T_{SCM} ($CD45RA^{+}CD28^{+}CCR7^{+}$), early T_{CM} ($CD45RA^{+}CD28^{-}CCR7^{+}$), late T_{CM}, also called transitional memory, ($CD45RA^{+}CD28^{-}CCR7^{-}$), T_{EM} ($CD45RA^{-}CD28^{-}CCR7^{-}$) and T_{EMRA} ($CD45RA^{+}CD28^{-}CCR7^{-}$) cells were analysed for the proportion that were CD57 bright. Cells had first been selected by FSC/SSC, CD3-positivity and CD8 expression. This plot shows $CD8^{+}$ T cells, a similar gating strategy was applied to $CD4^{+}$ T cells.

Supplementary Tables

CD8$^+$ T cells

	Uninfected			HCV			HTLV-1			HIV-1		
	KIR2DL1	KIR2DL2/L3	KIR3DL1	KIR2DL1	KIR2DL2/L3	KIR3DL1	KIR2DL1	KIR2DL2/L3	KIR3DL1	KIR2DL1	KIR2DL2/L3	KIR3DL1
Naïve/T_{SCM}	0.020	0.218	0.060	0.006	0.142	0.229	0.055	0.296	0.067	0.007	0.102	0.485
T_{CM}	0.036	0.671	0.204	0.005	0.339	0.207	0.011	0.952	0.166	0.011	0.406	0.424
T_{EM}	0.491	6.816	1.477	0.404	7.685	2.487	1.717	10.743	1.371	0.083	2.029	0.694
T_{EMRA}	0.806	21.963	3.963	0.272	10.041	3.080	0.635	52.782	6.357	0.369	4.082	1.801
BULK	0.150	4.560	1.160	0.079	3.330	0.876	0.012	16.714	1.187	0.114	1.666	0.787

CD4$^+$ T cells

	Uninfected			HCV			HTLV-1			HIV-1		
	KIR2DL1	KIR2DL2/L3	KIR3DL1	KIR2DL1	KIR2DL2/L3	KIR3DL1	KIR2DL1	KIR2DL2/L3	KIR3DL1	KIR2DL1	KIR2DL2/L3	KIR3DL1
Naïve/T_{SCM}	0.039	0.071	0.077	0.007	0.067	0.026	1.012	0.050	0.019	0.006	0.115	0.139
T_{CM}	0.049	0.303	0.098	0.006	0.135	0.134	1.119	0.158	0.043	0.006	0.075	0.078
T_{EM}	0.260	2.979	0.455	0.000	1.494	0.701	1.633	0.583	0.514	0.000	0.714	0.284
T_{EMRA}	0.214	2.511	0.447	0.111	1.188	0.374	2.077	2.561	1.267	0.071	12.626	2.362
BULK	0.047	0.259	0.096	0.007	0.121	0.084	0.008	0.143	0.039	0.008	0.321	0.103

Supp Table 1. Median iKIR expression (expressed as a percentage of the parent subpopulation) for different CD8$^+$ (top table) and CD4$^+$ (bottom table) T cell subpopulations (naïve/T_{SCM}, T_{CM}, T_{EM}, and T_{EMRA}) as well as total (bulk) T cells. If an individual is genetically negative for a given iKIR then that individual is excluded from the median calculations for that iKIR.

	Cohort 1		Cohort 2	
**Independent Variable**	**Coefficient (std. error)**	***P***	**Coefficient (std. error)**	***P***
Gender Male (vs. Female)	0.009 (0.005)	0.05	-	-
Subset CD8 (vs.CD4)	0.016 (0.005)	0.0007	0.008 (0.003)	0.0018
Differentiation Stage	0.016 (0.002)	$4x10^{-14}$	0.010 (0.001)	$4x10^{-17}$
KIR KIR2DL2/3 (vs. KIR2DL1)	0.04 (0.006)	$4x10^{-12}$	0.04 (0.003)	$7x10^{-35}$
KIR3DL1 (vs. KIR2DL1)	0.01 (0.006)	0.06	0.004 (0.003)	0.18

Supp Table 2. Linear multivariate model to identify predictors of iKIR expression on T cells. The full (starting) model was

iKIR expression ~ gender + T cell subset + age + CMV serostatus + cell differentiation stage + infection status + KIR

Dependent variable: proportion of the T cell population that expressed iKIR. Independent variables considered in base model: age, CMV serostatus, gender, cell differentiation stage (as an ordinal variable 1=naïve/T stem cell, 2=central memory, 3=effector memory, 4=effector memory RA revertant), T cell subset (as factor CD8 vs CD4), iKIR (as factor with KIR2DL1 as base). Non-significant variables were removed by forward and backward selection. Final models, for cohort 1 and cohort 2 respectively, were:

Cohort 1: iKIR expression ~ gender + T cell subset + cell differentiation stage + iKIR
Cohort 2 iKIR expression ~ T cell subset + cell differentiation stage + iKIR

Number of hypotheses tested =7; p value threshold for α=0.05 is P= 0.009 in cohort 1 and P=0.007 in cohort 2 (permutation test).

ID	f	se f	δ	se δ	b_w	se b_w
DW14	0.0353	0.0018	0.067	0.002	4.30	0.28
DW15	0.0314	0.0019	0.067	0.002	4.71	0.35
DW17	0.0235	0.0019	0.067	0.002	5.11	0.50
HC01	0.0274	0.0024	0.067	0.002	4.18	0.45
HC06	0.0316	0.0019	0.067	0.002	3.94	0.31
HC07	0.0255	0.0022	0.067	0.002	5.01	0.50
HC08	0.0298	0.002	0.067	0.002	3.89	0.34
HC09	0.0186	0.0019	0.067	0.002	5.35	0.62
HC11	0.0305	0.0019	0.067	0.002	4.87	0.36
HC12	0.0336	0.0024	0.067	0.002	5.23	0.42
HC13	0.0235	0.0018	0.067	0.002	5.30	0.49
HT01	0.0226	0.0022	0.067	0.002	4.94	0.58
HT02	0.0295	0.0019	0.067	0.002	5.08	0.39
HT03	0.0362	0.0019	0.067	0.002	5.36	0.33
HV01	0.0141	0.0018	0.067	0.002	4.80	0.75
HV02	0.0364	0.002	0.067	0.002	5.52	0.35
HV07	0.0245	0.0018	0.067	0.002	5.59	0.49
HV08	0.0276	0.0018	0.067	0.002	5.53	0.43
HV10	0.0329	0.0021	0.067	0.002	4.66	0.38
HV12	0.0229	0.002	0.067	0.002	6.17	0.53

Supp Table 3. Saliva and monocyte parameter estimates. Parameter estimates and standard errors of the saliva parameters f and δ and the monocyte parameter b_w for all individuals. f is the asymptote of label in the body water, δ is the rate at which this asymptote is approached (see equation 1 in main text, methods) and bw is the amplification factor (see equation 2 in main text, methods). The standard error of a parameter estimate is calculated as the standard deviation of 3000 samples of the posterior distribution of the corresponding parameter.

ID	Infection Status	Cells	iKIR expression	Functional iKIR count	*p*	se *p*	*d**	se *d**
DW14	Control	T_{CM}	Func	3	0.0057	0.0007	0.0142	0.0036
DW14	Control	T_{EMRA}	Func	3	0.0017	0.0004	0.0084	0.0054
DW14	Control	T_{EMRA}	Non Func	3	0.0030	0.0006	0.0118	0.0056
DW15	Control	T_{EMRA}	Non Func	4	0.0014	0.0005	0.0120	0.0094
DW17	Control	T_{CM}	KIR Neg	3	0.0059	0.0013	0.0225	0.0083
DW17	Control	T_{EMRA}	KIR Neg	3	0.0019	0.0006	0.0125	0.0096
DW17	Control	T_{EMRA}	Func	3	0.0020	0.0006	0.0112	0.0080
HC01	HCV	T_{CM}	Func	3	0.0021	0.0008	0.0139	0.0102
HC01	HCV	T_{EMRA}	Func	3	0.0017	0.0006	0.0135	0.0101
HC06	HCV	T_{CM}	Func	2	0.0038	0.0009	0.0137	0.0069
HC06	HCV	T_{CM}	Non Func	2	0.0023	0.0007	0.0133	0.0093
HC06	HCV	T_{EMRA}	Func	2	0.0032	0.0007	0.0093	0.0052
HC06	HCV	T_{EMRA}	Non Func	2	0.0027	0.0006	0.0093	0.0056
HC07	HCV	T_{CM}	KIR Neg	2	0.0017	0.0005	0.0118	0.0087
HC07	HCV	T_{CM}	Func	2	0.0021	0.0005	0.0110	0.0069
HC07	HCV	T_{EMRA}	KIR Neg	2	0.0028	0.0005	0.0079	0.0045
HC07	HCV	T_{EMRA}	Func	2	0.0031	0.0006	0.0062	0.0042
HC08	HCV	T_{CM}	KIR Neg	3	0.0024	0.0007	0.0123	0.0083
HC08	HCV	T_{EMRA}	KIR Neg	3	0.0023	0.0006	0.0102	0.0068
HC08	HCV	T_{EMRA}	Func	3	0.0024	0.0006	0.0100	0.0068
HC09	HCV	T_{CM}	KIR Neg	2	0.0031	0.0009	0.0142	0.0082
HC09	HCV	T_{CM}	Func	2	0.0021	0.0009	0.0172	0.0142
HC09	HCV	T_{EMRA}	KIR Neg	2	0.0036	0.0008	0.0072	0.0044
HC09	HCV	T_{EMRA}	Func	2	0.0049	0.0008	0.0073	0.0037
HC11	HCV	T_{EMRA}	Non Func	4	0.0045	0.0006	0.0079	0.0035
HC12	HCV	T_{EMRA}	Func	2	0.0027	0.0005	0.0076	0.0039
HC13	HCV	T_{CM}	Non Func	2	0.0023	0.0007	0.0131	0.0080
HC13	HCV	T_{EMRA}	Func	2	0.0043	0.0007	0.0071	0.0037
HC13	HCV	T_{EMRA}	Non Func	2	0.0042	0.0007	0.0064	0.0036
HT01	HTLV-1	T_{EMRA}	KIR Neg	2	0.0043	0.0007	0.0082	0.0038
HT01	HTLV-1	T_{EMRA}	Func	2	0.0048	0.0008	0.0085	0.0039
HT01	HTLV-1	T_{EMRA}	Non Func	2	0.0050	0.0009	0.0110	0.0048
HT02	HTLV-1	T_{CM}	KIR Neg	2	0.0110	0.0012	0.0290	0.0044
HT02	HTLV-1	T_{CM}	Func	2	0.0064	0.0010	0.0189	0.0049
HT02	HTLV-1	T_{EMRA}	KIR Neg	2	0.0080	0.0009	0.0096	0.0028
HT02	HTLV-1	T_{EMRA}	Func	2	0.0056	0.0007	0.0076	0.0031
HT03	HTLV-1	T_{CM}	Func	3	0.0019	0.0004	0.0106	0.0055
HT03	HTLV-1	T_{EMRA}	Func	3	0.0033	0.0004	0.0059	0.0025
HV01	HIV-1	T_{CM}	Func	2	0.0092	0.0031	0.0340	0.0143
HV01	HIV-1	T_{EMRA}	Func	2	0.0070	0.0015	0.0114	0.0054

ID	Infection Status	Cells	iKIR expression	Functional iKIR count	*p*	se *p*	*d**	se *d**
HV01	HIV-1	T_{EMRA}	Non Func	2	0.0060	0.0012	0.0087	0.0047
HV02	HIV-1	T_{EMRA}	Func	2	0.0028	0.0004	0.0064	0.0032
HV02	HIV-1	T_{EMRA}	Non Func	2	0.0066	0.0006	0.0098	0.0025
HV07	HIV-1	T_{CM}	KIR Neg	4	0.0056	0.0011	0.0103	0.0083
HV07	HIV-1	T_{CM}	Func	4	0.0062	0.0012	0.0117	0.0086
HV07	HIV-1	T_{EMRA}	KIR Neg	4	0.0024	0.0006	0.0117	0.0097
HV07	HIV-1	T_{EMRA}	Func	4	0.0029	0.0008	0.0116	0.0111
HV08	HIV-1	T_{EMRA}	Func	2	0.0071	0.0009	0.0100	0.0035
HV08	HIV-1	T_{EMRA}	Non Func	2	0.0068	0.0009	0.0106	0.0035
HV10	HIV-1	T_{CM}	Func	3	0.0155	0.0018	0.0363	0.0053
HV10	HIV-1	T_{EMRA}	Func	3	0.0035	0.0006	0.0085	0.0043
HV10	HIV-1	T_{EMRA}	Non Func	3	0.0059	0.0011	0.0123	0.0050
HV12	HIV-1	T_{CM}	KIR Neg	3	0.0069	0.0008	0.0038	0.0023
HV12	HIV-1	T_{CM}	Func	3	0.0070	0.0008	0.0048	0.0024
HV12	HIV-1	T_{EMRA}	KIR Neg	3	0.0030	0.0007	0.0052	0.0043
HV12	HIV-1	T_{EMRA}	Func	3	0.0031	0.0007	0.0054	0.0045

Supp Table 4. $CD8^+$ T cell parameter estimates. Parameter estimates and standard errors of the T lymphocyte parameters *p* and *d** for all cell populations and all individuals. *p* is the average rate of proliferation and *d** is the rate of disappearance of labelled cells (see equation 3 in main text methods). The standard error of a parameter estimate is calculated as the standard deviation of 3000 samples of the posterior distribution of the corresponding parameter. The column "iKIR expression" denotes the iKIR expression status of the sorted T cell population where "Func" denotes expression of a functional iKIR, "non Func" denotes expression only of nonfunctional iKIR and "KIR Neg" means the sorted $CD8^+$ T cell population was iKIR-negative. Functional iKIR count is the count of iKIR-ligand gene pairs in the genome for that individual. DW15 and HC11 were positive for classical KIR3DL2 ligands (HLA-A*03, -A*11) and so were excluded from the first round of analysis (see results and methods). Cell lifespan is defined as *1/p*; it is the average time between creation of a cell (by proliferation) and loss of the cell (by proliferation, death, differentiation); it differs from half-life by a factor of ln(2) i.e. half life = ln(2).lifespan.

	Independent Variable	Coefficient (std. error)	*P*
**A.** Inclusion of KIR expression status	Infection status (vs. uninfected)		
	HCV	0.91 (0.29)	0.003
	HIV	-0.32 (0.31)	0.3
	HTLV	0.11 (0.34)	0.8
	Cell subpopulation (vs. CM)		
	TEMRA	1.03 (0.32)	0.003
	Infection status:cell subpopulation		
	HCV:TEMRA	-1.29 (0.36)	0.001
	HIV:TEMRA	-0.43 (0.37)	0.262
	HTLV:TEMRA	-0.98 (0.42)	0.024
	iKIR expression status (vs. negative+ nonfunctional)		
	functional	0.1 (0.1)	0.337
**B.** Inclusion of functional iKIR gene count	Infection status (vs. uninfected)		
	HCV	1.22 (0.24)	$7x10^{-6}$
	HIV	-0.37 (0.24)	0.13
	HTLV	0.4 (0.28)	0.16
	Cell subpopulation (vs. CM)		
	TEMRA	1.03 (0.26)	$2x10^{-4}$
	Infection status:cell subpopulation		
	HCV:TEMRA	-1.29 (0.29)	$5x10^{-5}$
	HIV:TEMRA	-0.24 (0.3)	0.43
	HTLV:TEMRA	-0.93 (0.33)	0.01
	Functional iKIR gene count	0.41 (0.08)	$3x10^{-6}$

Supp Table 5. Linear multivariate model to identify predictors of CD8$^+$ T cell lifespan. The independent variable (T cell lifespan, *1/p*) was natural log transformed as this resulted in a more linear relationship with the covariates. Inclusion of an interaction term between cell population and infection status performed significantly better than a model without an interaction term (ANOVA, P=0.001); this was because all three virus infections significantly increased proliferation of T_{EMRA} but not of T_{CM}. So, the base model was of the form

Log(lifespan) ~ (infection status)*(cell subpopulation)

A Inclusion of KIR expression status. Coefficients and P values shown for the base model with inclusion of the iKIR expression status of the T cell (negative and nonfunctional grouped and treated as baseline).

B Inclusion of functional iKIR count. Coefficients and P values shown for the base model with inclusion of the count of functional iKIR genes in the individual.

Number of hypotheses tested =2 (iKIR expression status is correlated with lifespan, functional iKIR count is correlated with lifespan).

	CD8$^+$ T cells		CD4$^+$ T cells	
**Independent Variable**	**Coefficient (std. error)**	***P***	**Coefficient (std. error)**	***P***
Age (years)	0.003 (0.001)	0.07	-	
CMV Infection Status:				
Positive (vs. Negative)	-		0.06 (0.02)	0.01
Differentiation Stage	0.15 (0.007)	$<2x10^{-16}$	0.09 (0.008)	$<2x10^{-16}$
Number of functional iKIR	0.03 (0.01)	0.003	0.02 (0.01)	0.1
Adjusted R2	0.59		0.30	

Supp Table 6. Linear multivariate model to identify predictors of CD57 expression. Dependent variable: proportion of the T cell subpopulation that was CD57 bright. Independent variables considered in base model: Age, CMV serostatus, gender, cell differentiation stage (as an ordinal variable 1=naïve/stem cell like memory, 2=early central memory, 3=late central memory, 4=effector memory, 5=effector memory RA revertant), functional iKIR count. Non-significant variables (with the exception of functional iKIR count which we wanted to explicitly test) were removed. Final models, for CD8$^+$ and CD4$^+$ T cells respectively, were:

$$CD8: \quad CD57 \sim Age + Differentiation\, stage + func\, iKIR\, count$$

$$CD4: \quad CD57 \sim CMV\ status + Differentiation\, stage + func\, iKIR\, count$$

Number of hypotheses tested=1 (functional iKIR count is positively correlated with CD57 expression).

	$CD8^+$ T cells		$CD4^+$ T cells	
**Independent Variable**	**Coefficient (std. error)**	***P***	**Coefficient (std. error)**	***P***
Age (years)	0.007 (0.003)	0.009	-	
CMV Infection Status:				
Positive (vs. Negative)	-		0.14 (0.05)	0.007
Number of functional iKIR	0.078 (0.022)	0.0005	0.044 (0.027)	0.10

Supp Table 7. Linear multivariate model to identify predictors of CD57 expression in late stage differentiated cells. As in Supp Table 6 but naïve and central memory T cells (which express very low levels of CD57) were excluded from the analysis. Final models, for $CD8^+$ and $CD4^+$ T cells respectively, were:

$$CD8: \quad CD57 \sim Age + func\,iKIR\,count$$
$$CD4: \quad CD57 \sim CMV\ status + func\,iKIR\,count$$

Supplementary Methods

Flow cytometry panels

KIR expression analysis and stable isotope labelling (cohort 1)

Product	Supplier	Product code
eBioscience™ Fixable Viability Dye eFluor™ 780	Thermofisher Scientific	65-0865-18
Ultracompbeads 100 tests	Thermofisher Scientific	01-2222-42
APC/Cy7 anti-human CD4 Antibody	Biolegend	317418
APC/Cy7 anti-human CD14 Antibody	Biolegend	367108
APC anti-human CD158b (KIR2DL2/L3, NKAT2)	Biolegend	312612
Brilliant Violet 421™ anti-human CD28	Biolegend	302930
Percp-Cy5.5 Mouse anti-human CD45RA	Biolegend	304122
PE/Cy7 anti-human CD56 (NCAM)	Biolegend	318318
Brilliant Violet 510™ anti-human CD16	Biolegend	302048
APC-Cy7Mouse anti-human CD19	Biolegend	363010
PE anti-human CD158e1 (KIR3DL1, NKB1)	Biolegend	312708
FITC anti-human CD14	Biolegend	301804
FITC Human KIR2DL1/KIR2DS5	Bio-Techne	FAB1844F
Alexa Fluor® 488 Human KIR2DL1/KIR2DS5	Bio-Techne	FAB1844G
APC Human KIR3DL2/CD158k	Bio-Techne	FAB2878A
PE Human KIR3DL2/CD158k	Bio-Techne	FAB2878P
Alexa Fluor® 488 Human KIR3DL2/CD158k	Bio-Techne	FAB2878G
ECD-Mouse anti-human CD8	Beckman Coulter	B08467
Alexa Fluor® 700 Mouse Anti-Human CD3	BD Diagnostics	557943

KIR expression analysis independent replication (cohort 2)

Antibody	Supplier	Product code
PE-Cyanine7 mouse anti-human CD3	Invitrogen	25-0037-42
APC/Fire 750 (SK3) mouse anti-human CD4	Biolegend	980814
APC/Fire 750 (SK1) mouse anti-human CD8	Biolegend	980914
Alexa Flour 488 (H100) mouse anti-human CD45RA	Biolegend	304114
PerCP-Cy5.5 mouse anti-human CD28	Invitrogen	45-0289-42
KIR2DL1-PE (REA284)	Miltenyi Biotec	130-120-586
PE mouse anti-human KIR2DL2/3-PE (DX27)	Biolegend	312606
PE mouse anti-human CD158e1 (KIR3DL1, NKB1)	Biolegend	312708

CD57 expression analysis (cohort 3)

**Antibody**	**Supplier**	**Product code**
PE-Cyanine7 mouse anti-human CD3	Invitrogen	25-0037-42
V500 mouse anti-human CD4	BD Diagnostics	560768
APC-Cy7 mouse anti-human CD8	BD Diagnostics	557760
PerCP-Cy5.5 mouse anti-human CD28	Invitrogen	45-0289-42
APC mouse anti-human CD45RA	Invitrogen	17-0458-42
PE mouse anti-human CCR7	Bio-Techne	FAB197P

Definition of count of functional iKIR genes

We used our previous definition of count of functional iKIR genes(1, 2) since our aim was to investigate possible mechanisms underlying our prior observation that functional iKIR count enhanced HLA associations with clinical outcome. This measure only included the iKIR known to bind HLA class I alleles and was based on iKIR presence or absence without taking into account iKIR allele level information. Thus, the number of iKIR gene-ligand pairs carried by an individual was counted using the following definition of a ligand:

**iKIR**	**ligand**
KIR2DL1	C2
KIR2DL2	C1 (including HLA-B*46, -B*73) and C2
KIR2DL3	C1 (including HLA-B*46, -B*73)
KIR3DL1	Bw4 (including HLA-A*23, -A*24, -A*32 and some other rare HLA A alleles)

Where C1 denotes an HLA allele carrying a C1 motif (Asparagine at position 80); C2 an HLA allele with a C2 motif (Lysine at position 80) and Bw4 an HLA allele with a Bw4 motif (Asparagine at position 77).

Supplementary Results

Supplementary results 1. Expression of a functional iKIR is not a significant predictor of CD8$^+$ T cell lifespan.

We performed multivariate regression to test if iKIR expression status predicted the T cell lifespan with covariates: cell subpopulation (T_{CM} or T_{EMRA}) and infection status (HIV-1 /HCV /HTLV-1 /uninfected). The independent variable (T cell lifespan) was log transformed (base e) as this resulted in a more linear relationship with the covariates. Studying the covariates (in a model without iKIR expression status as a variable) we found that inclusion of an interaction term between cell population and infection status performed significantly better than a model without an interaction term (ANOVA, P=0.001); this was because all three virus infections significantly increased proliferation of T_{EMRA} but not of T_{CM}. So going forward our base model was of the form

$\mathrm{Ln}(p^{-1}) \sim$ (infection status)*(cell subpopulation)

Note that as cell lifespan =1/p and ln(p)= -ln(1/p) all results are identical, up to sign of the coefficient, whether the independent variable is ln(p) or ln(lifespan). Including KIR expression status (functional or non-functional/KIR negative) did not improve the performance of the model (ANOVA, no evidence to reject the base model P=0.34) and the dependent variable KIR expression status was not significant (Est = +0.1 P=0.34). Coefficients with standard error and P values of all model covariates are provided in **Supp Table 5A**.

We conclude that expression of one or more functional iKIR is not a significant predictor of T cell lifespan.

Supplementary results 2. Count of functional iKIR genes is a highly significant predictor of CD8$^+$ T cell lifespan.

The variable, count of functional iKIR, was added to the base model. It significantly improved model performance and the variable itself was highly statistically significant (Est= +0.41, P= 3.4×10^{-6}). Coefficients with standard error and P values of all model covariates are provided in **Supp Table 5B**.

Connecting the estimate of the coefficient to the change in lifespan. The median ln(lifespan) of CD8$^+$ T cells in this dataset for individuals with 2 functional iKIR genes (after adjusting for other variables) is 4.74 so a difference in functional iKIR count from 2 to 3 results in an increase in lifespan of exp(4.74 + 0.41) - exp(4.74) = 58 days and a difference in functional iKIR count from 2 to 4 results in an increase in lifespan of exp(4.74 + 0.41*2) - exp(4.74) = 145 days. A similar (but less precise) estimate is obtained when the predicted variable is lifespan rather than log(lifespan): here the coefficient (now directly interpretable as the change in

lifespan for every unit change in count of functional iKIR) is 105 days ($P=2.2x10^{-5}$). Although the coefficients of this model are easier to interpret it is not statistically preferred as taking the logs increases the linearity of the relationship between lifespan and the covariates; though we note that the conclusions that count of functional iKIR genes is a highly significant predictor of $CD8^+$ T cell lifespan with an increase of the order of approximately 2 months for each increase in functional iKIR gene is the same whichever model is used.

Supplementary Results 3. Which is the best predictor: count of functional iKIR genes, inhibitory KIR score or count of iKIR genes?

We have previously also used an inhibitory KIR score as a predictor(1, 2). The score is similar to the count of iKIR-ligand pairs but is weighted to reflect the fact that some iKIR-ligand interactions are known to be stronger than others (i.e. KIR2DL2 binds C1 more strongly on average than it binds C2, KIR2DL2 binds C1 more strongly on average than KIR2DL3 binds C1). Functionally diverse alleles at the same locus (2DL2/L3 and 3DL1/S1) were scored differently to reflect different strengths of inhibitory signal. As previously, the following definition is used:

> Inhibitory score= (1 if *KIR2DL1* & *C2*) + (1 if *KIR2DL2* & *C1, B46, B73* or 0.5 if *KIR2DL2* with only *C2*) + (0.75 if *KIR2DL3* & *C1, B46, B73*) + (1 if *KIR3DL1* and *Bw4*).

Inhibitory score and count of functional iKIR are highly correlated and both are significant predictors of T cell lifespan and both significantly impact HLA class I associations. Functional iKIR count performs slightly better in predicting lifespan but the difference between the models is not significant (P=0.09, Davidson-MacKinnon J test(3)). We also consider the number of unique iKIR genes that an individual carried (i.e. without considering presence or absence of the HLA ligand). Again, this is correlated with the count of functional iKIR but it was a poor predictor of lifespan and the model with the count of functional iKIR performed significantly better ($P=9x10^{-5}$, Davidson-MacKinnon J test); indicating that ligation of the iKIR is essential for the increase in T cell lifespan.

Supplementary Results 4. Increase in KIR^+ T cell lifespan required to explain the bulk effect.

We see a doubling in bulk $CD8^+$ T cell lifespan as the functional iKIR count changes from 2 to 4. For this to be explained by an increase in lifespan just of the small proportion (6%) of $CD8^+$ T cells that express iKIR then the lifespan (*x*) of $iKIR^+CD8^+$ T cells would need to increase by a fraction *f* where

$$\frac{6}{100} fx + \frac{94}{100} x = 2x$$

Rearranging

$$f = \frac{2x - 0.94x}{0.06x}$$
$$= 17.6$$

That is the lifespan of $iKIR^{+}CD8^{+}$ T cells would need to increase by 17.6 -fold to explain the bulk effect.

Supplementary Results 5. Derivations for Model predictions of the relationship between functional iKIR gene count and $CD8^{+}$ T cell age

Consider the model given by equation 4 in the main text methods. If $\frac{d\bar{z}}{d\mu} < 0$ for all positive values of μ, s and λ, and we assume that increasing functional iKIR gene count increases CD8+ T cell lifetime (decreases μ), then increasing functional iKIR gene count increases the CD8+ T cell count at equilibrium $\bar{z}$.

Solving for $\bar{z}$ by setting $\frac{dz}{dt} = 0$ yields

$$0 = \lambda + s\bar{z}\left(1 - \frac{\bar{z}}{k}\right) - \mu\bar{z}$$

Differentiating both sides with respect to μ and rearranging yields

$$\frac{d\bar{z}}{d\mu} = \frac{\bar{z}^{\,2}}{-\lambda - \frac{s\bar{z}^2}{k}}$$

Which is less than 0 for all positive parameter values.

Multiplying $D = \mu - s + \frac{s\bar{z}}{k}$ by $\bar{z}$ on both sides yields $D\,\bar{z} = \mu\bar{z} - s\bar{z} + \frac{s\bar{z}^2}{k} = \lambda$, so $\bar{z} = \frac{\lambda}{d}$.

References for Supplementary Material

1. Boelen L, et al. Inhibitory killer cell immunoglobulin-like receptors strengthen CD8(+) T cell-mediated control of HIV-1, HCV, and HTLV-1. *Sci Immunol.* 2018;3(29).
2. Seich Al Basatena NK, et al. KIR2DL2 enhances protective and detrimental HLA class I-mediated immunity in chronic viral infection. *PLOS Pathog.* 2011;7(10):e1002270.
3. Davidson R, and Mackinnon JG. *Several tests for model specification in the presence of alternative hypotheses.* Kingston, Ont.: Institute for Economic Research, Queens University; 1980.
